# Analysis of 200 unrelated individuals with a constitutional *NF1* deep intronic pathogenic variant reveals that variants flanking the alternatively spliced *NF1* exon 31 [23a] cause a classical neurofibromatosis type 1 phenotype while altering predominantly *NF1* isoform type II

**DOI:** 10.1007/s00439-023-02555-z

**Published:** 2023-04-25

**Authors:** Magdalena Koczkowska, Yunjia Chen, Jing Xie, Tom Callens, Alicia Gomes, Katharina Wimmer, Ludwine M. Messiaen

**Affiliations:** 1 Medical Genomics Laboratory, Department of Genetics, University of Alabama at Birmingham, Birmingham, AL 35294 USA; 2 3P-Medicine Laboratory, Medical University of Gdansk, 80-211 Gdansk, Poland; 3 Institute of Human Genetics, Medical University of Innsbruck, 6020 Innsbruck, Austria; 4 Present Address: Natera, Inc., San Carlos, CA USA

## Abstract

Neurofibromatosis type 1 results from loss-of-function *NF1* pathogenic variants (PVs). Up to 30% of all *NF1* PVs disrupt mRNA splicing, including deep intronic variants. Here, we retrospectively investigated the spectrum of *NF1* deep intronic PVs in a cohort of 8,090 unrelated individuals from the University of Alabama at Birmingham (UAB) dataset with a molecularly confirmed neurofibromatosis type 1. All variants were identified through their effect on the *NF1* transcript, followed by variant characterization at the DNA-level. A total of 68 distinct variants, which were ≥ 20 nucleotides away from the closest exon–intron junction, were identified in 2.5% unrelated individuals with NF1 (200/8,090). Nine different pathogenic splice variants, identified in 20 probands, led to exonization of different parts of intron 30 [23.2] or 31 [23a]. The two major *NF1* transcript isoforms, distinguished by the absence (type I) or presence (type II) of the alternatively spliced cassette exon 31 [23a], are equally expressed in blood in control individuals without NF1 or NF1-affected individuals carrying their PV not in the introns flanking exon 31 [23a]. By fragment and cloning analysis we demonstrated that the exonization of intron 31 [23a] sequences due to deep intronic PV predominantly affects the *NF1* isoform II. Seven additional (likely) pathogenic *NF1* deep intronic variants not observed in the UAB dataset were found by classification of 36 variants identified by a literature search. Hence, the unique list of these 75 deep intronic (likely) PVs should be included in any comprehensive *NF1* testing strategy.

**Supplementary Information:**

The online version contains supplementary material available at 10.1007/s00439-023-02555-z.

## Introduction

Precise pre-mRNA splicing is critical for functional protein translation. Proper splicing requires the accurate recognition of exon–intron boundaries, i.e. the splice sites, and other regulatory sequences, such as exonic and intronic splice enhancers and silencers (Cartegni et al. 2002). Some variants at these sequences, such as point variants, may result in improper intron removal and subsequent alteration of the open reading frame. However, also variants in introns may affect proper splicing, most commonly by creating a novel splice acceptor or donor site that leads to exonization of the intronic sequences between this novel and a corresponding pre-existing cryptic intronic splice donor or acceptor site (splice variants type II according to Wimmer et al. 2007; details in Figure S1). Other disease-causing mechanisms of deep intronic variants include the use of a novel instead of the natural splice site (splice variants type III according to Wimmer et al. 2007; details in Figure S1), disruption of splice enhancers or silencers and/or inactivation of non-coding RNA genes (Vaz-Drago et al. 2017). Genomic rearrangements involving deep intronic sequences occur very rarely, however a few reports for the *DMD* gene (MIM *300377) have been published (Baskin et al. 2011; Khelifi et al. 2011 with references therein).

Loss-of-function variants in the *NF1* tumor suppressor gene (MIM *613113) cause neurofibromatosis type 1 (NF1, MIM #162200), which has one of the highest spontaneous mutation rates across all monogenic human disorders, described as up to 1 × 10^–4^ (Huson et al. 1989). Considering in addition the large size of the gene, including 57 exons and three alternatively spliced exons, the wide *NF1* allelic heterogeneity is not surprising. To date, a total of 3,748 and 3,375 unique *NF1* (likely) pathogenic variants (PVs) were deposited in the Leiden Open Variation Database (LOVD) and ClinVar databases, respectively (as of 17^th^ of June, 2022). Several studies demonstrated that roughly 30% of the *NF1* variants affect mRNA splicing, but only a third of these affect the GT-AG dinucleotides at the canonical splice sites (Messiaen et al. 2000; Wimmer et al. 2007, 2020; Messiaen and Wimmer 2012). *NF1* deep intronic PVs, altering the mRNA splicing, were previously described in the NF1-affected individuals, but the full spectrum of such *NF1* variants has not been investigated yet.

The *NF1* gene encodes neurofibromin, a GTPase activating protein (GAP) downregulating the RAS signal transduction pathway through its GAP-related domain (GRD), one of the best characterized functional domains of neurofibromin (Ballester et al. 1990; DeClue et al. 1991). The alternatively spliced exon 31 [23a] is located within the GRD. The two most common *NF1* isoforms either lack or include exon 31 [23a] (isoform type I and type II, respectively) (Uchida et al. 1992; Andersen et al. 1993). Both isoforms are equally expressed in blood of the NF1-affected patients and healthy controls, whereas isoform I is predominantly expressed in the central nervous system. *NF1* isoform I encodes the GRD of neurofibromin with lower affinity to Ras-GTP, but ten times higher Ras-GTPase activity than isoform II. Therefore, it is believed that the alternative splicing of exon 31 [23a] plays a significant role in regulating the GTPase activity as well as the Ras signaling pathway. In order to further elucidate the function of exon 31 [23a], Costa et al. (2001) generated a mouse model with biallelic knock out of exon 31 [23a]. The Nf1^23a−/−^ mice are viable, develop normally and do not have an increased tumor predisposition, however, present a disability in spatial learning and memory. These findings suggest that alternative splicing of exon 31 [23a] might be critical for brain functions.

Targeted DNA-based next-generation sequencing (NGS) has now been integrated in routine clinical genetic testing in many laboratories, typically including the protein-coding gene sequences along with ± 20–30 base pairs (bp) of flanking intronic sequences. Of note, the regions harboring deep intronic variants are typically not covered by current NGS panels. For many years, before transitioning to an NGS-based approach, the Medical Genomics Laboratory at the University of Alabama at Birmingham (UAB) used RNA-based protocols as the first-tier *NF1* variant detection test, which allowed to identify deep intronic *NF1* variants affecting splicing by their effect on the transcript and further characterization at the DNA-level as previously described (Messiaen et al. 2000; Messiaen and Wimmer 2012). In the current study, we retrospectively summarized the spectrum and prevalence of *NF1* deep intronic PVs, residing ≥ 20 nucleotides away from the closest exon–intron junction, in a large cohort of 8,090 unrelated individuals from the UAB dataset with a molecularly confirmed NF1. Importantly, we demonstrate that deep intronic variants residing either 5′ or 3′ of exon 31 [23a] predominantly affect *NF1* the isoform II. Furthermore, we compiled a unique list of 75 deep intronic (likely) PVs, including 68 variants identified in the UAB dataset and an additional seven found through a comprehensive review of the current literature and publicly available databases. The intronic loci in which these variants reside should be included in any comprehensive *NF1* testing strategy.

## Patients and methods

### Individuals and phenotypic data

A total of 200 unrelated individuals heterozygous for a deep intronic (likely) PV in the *NF1* gene out of 8,090 *NF1* (likely) PVs-positive probands were included in this study. Samples were originally referred to the Medical Genomics Laboratory at UAB for *NF1* clinical genetic testing to establish or confirm the diagnosis of NF1 through 2003–2018. For all individuals the phenotypic checklist form, as previously reported (Rojnueangnit et al. 2015; Koczkowska et al. 2018, 2019, 2020), was submitted at the time when genetic testing was ordered. For the current study, we used the clinical data as originally submitted and did not recontact referring physicians to request updated phenotypic information. Individuals with missing data for a particular clinical symptom were classified as “unknown” or “not specified” and consequently excluded from that part of the aggregated clinical data.

### Comprehensive *NF1* molecular analysis

Comprehensive *NF1* variant analysis using an RNA-based assay complemented by DNA-dosage analysis by multiplex ligation-dependent probe amplification (MLPA) and fluorescence *in-situ* hybridization (FISH) was performed as previously described (Messiaen et al. 2000; Messiaen and Wimmer 2012). In this report, all the splicing defects with exonized intronic sequences were firstly detected by the direct cDNA sequencing of the entire *NF1* coding region. To prevent nonsense-mediated mRNA decay (NMD), RNA for cDNA sequencing was extracted from puromycin treated short-term lymphocyte cultures. Subsequently, the specific deep intronic (likely) PVs were identified and validated by genomic DNA (gDNA) sequencing using primers specifically designed to flank the affected intron regions. The nomenclature of the variants was based on the NM_000267.3 *NF1* messenger RNA (mRNA) reference sequence according to the Human Genome Variation Society guidelines, except for the variants residing in the introns flanking the alternatively spliced exon 31 [23a]. These variants were all described based on both NM_000267.3 and NM_001042492.2. For exon numbering, the NCBI numbering followed by the legacy numbering, originally developed by the NF1 community, in square brackets was applied (Messiaen and Wimmer 2012). The variants were classified based on the American College of Medical Genetics and Genomics and the Association for Molecular Pathology recommendations (Richards et al. 2015).

### In-silico analysis of splice sites, branch points and regulatory motifs

A number of *in-silico* tools and algorithms have been developed to predict the effect of variants on mRNA splicing based on the consensus splice motifs. To better understand the mechanisms underlying the mis-splicing caused by deep intronic variants, we conducted a comprehensive *in-silico* analysis. Namely, we evaluated the strength of all the wild-type splice sites, the de novo splice sites created by the variants and the cryptic splices sites activated by these variants using the following *in-silico* splicing predictions programs, i.e. NNSplice (v.0.9), MaxEntScan, SpliceSiteFinder-like and GeneSplicer, all embedded in Alamut Visual Plus™ software v.1.6.1 (SOPHiA GENETICS™) and SpliceAI.

SpliceAI (https://spliceailookup.broadinstitute.org/) is a splice site prediction software based on a 32-layer deep neural network (Jaganathan et al. 2019). The settings for SpliceAI were as follows: hg19 genome version, raw score type, max distance of 250 for those with the length of the affected sequence < 250 or 500 for others, without the usage of Illumina's pre-computed scores. Given that SpliceAI only provides one predicted position for each of Δ scores (Acceptor Loss, Donor Loss, Acceptor Gain, Donor Gain) at one setting, an “not available” (“N/A”) will be assigned if no correct prediction was made. NNSplice is a splice site prediction program based on neural networks (Reese et al. 1997). The default thresholds for the 5’ and 3’ splice sites were 0.4. The range of the score was between [0,1] with a higher score indicating a higher probability that the sequence was recognized as a splice site by the spliceosome. MaxEntScan is a method based on the Maximum Entropy Modeling of the short sequences in pre-mRNA splicing (Yeo and Burge 2004). The default threshold for both 5’ and 3’ splice sites was 0, and scores below 0 suggested non-canonical splice sites. The threshold of GeneSplicer (Pertea et al. 2001), a tool also dedicated to detect splice sites, was 0 similar as for MaxEntScan. SpliceSiteFinder-like (SSF-like) is based on Position Weight Matrices, computed from a set of constitutive sequences at the exon – intron junction at both 5’ and 3’ splice sites (Shapiro et al. 1987; Zhang et al. 2018). In order to reduce false positives, the threshold as 70 with [0,100] scoring range was defaulted. In addition, the new branch points created by deep intronic variants were evaluated with the branch point prediction tool integrated in Alamut Visual Plus™ software v.1.6.1 based on the matrices described by Zhang (1998).

### Splicing pattern analysis and fragment analysis

The exonized cryptic introns caused by deep intronic variants flanking exon 31 [23a] were further investigated by fragment analysis to examine whether the exonized introns were mis-spliced in both the *NF1* transcripts I and II or only in one of them. cDNA prepared from the affected individuals was amplified by the forward primer (5′-GTTAGAACCATCAGAGAGCCTT-3′) and the reverse primer (5′-CTTTGACATTAACTTCAAGCCC-3′) located in exon 30 [23.2] and exon 32 [24], respectively, using Platinum® Taq DNA Polymerase Kit (Invitrogen, Carlsbad, CA). The 5′ of the reverse primer was labeled with *FAM* fluorescence. The wild-type *NF1* transcripts excluding and including exon 31 [23a] were amplified into 295 bp and 358 bp fragments, respectively. The PCR products were further analyzed by fragment analysis and capillary electrophoresis on a 3730 DNA analyzer (Applied Biosystems), with the results analyzed by GeneMapper™ 4.0 (Applied Biosystems).

### Cloning analysis

To confirm and characterize the specific sequence of all types of splice products caused by the deep intronic variants flanking the alternatively spliced *NF1* exon 31 [23a] identified in the affected individuals, in addition to the wild-type products, cDNA samples were amplified by the forward primer (5′-GTTAGAACCATCAGAGAGCCTT-3′) and the reverse primer (5′-CTTTGACATTAACTTCAAGCCC-3′), located in exon 30 [23.2] and 32 [24], respectively, without *FAM* florescent label, using Platinum® Taq DNA Polymerase Kit (Invitrogen, Carlsbad, CA). The PCR products were subcloned according to the manufacturer’s instructions into the TOPO®-TA cloning® vector pCR 4-TOPO (Invitrogen). For each individual, ~ 80–110 clones were picked and sequenced. The splicing patterns were analyzed by reading the sequences directly in the Sequence Analysis (Applied Biosystems).

### Research of deep intronic variants reported in the literature

The publicly available databases, i.e. LOVD, the Human Gene Mutation Database (HGMD) and ClinVar (as of 16th of May, 2022), were searched for reports of NF1-affected individuals carrying any *NF1* deep intronic variants. For records filtering, we used the same definition of a deep intronic variant as in the UAB dataset, i.e. any variant residing ≥ 20 nucleotides away from the closest exon–intron boundary.

### Statistical analysis

For univariate analysis, two-tailed Fisher’s exact test with P < 0.05 considered as statistically significant was applied to compare categorical variables. These statistical analyses were performed with GraphPad software.

## Results

### Overview of (likely) pathogenic deep intronic variants reported in the UAB dataset

Combining direct cDNA and gDNA sequencing, a total of 68 different *NF1* deep intronic (likely) PVs in 200 unrelated individuals (2.47%, 200/8,090) were uncovered (Table S1). Two of these variants, i.e. NM_000267.3:c.4111-679G > A and NM_000267.3:c.5750-1178_5750-1163del reported in UAB-R2603 and UAB-R2054, respectively, were proven mosaic with the approximate fraction of 20–35% of reads. The three most prevalent variants were NM_000267.3:c.5749 + 332A > G (r.5749_5750ins5749 + 155_5749 + 331), NM_000267.3:c.1260 + 1604A > G (r.1260_1261ins1260 + 1605_1260 + 1646) and NM_000267.3:c.1642-449A > G (r.1642_1643ins1642-448_1642-1), together accounting for 33.5% (67/200) of the deep intronic variants, i.e. 16% (32/200), 11.5% (23/200) and 6% (12/200), respectively. These variants are also frequently reported in the publicly available databases (Table S2). Among the remaining 65 less prevalent variants, 25 are reported as well in the LOVD, ClinVar and/or HGMD databases (details in Table S1).

**Fig. 1:**
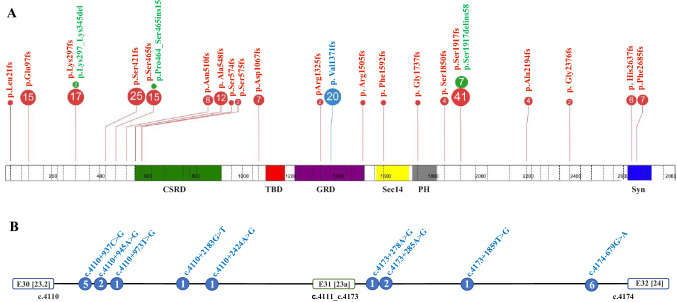
Spectrum of (likely) pathogenic *NF1* deep intronic variants in the studied cohort of 200 unrelated individuals from the UAB dataset. The upper panel (panel **A**) presents the list of 21 specific amino acids affected by 68 different deep intronic variants, resulting in frameshift (in red) or other effect (in green). The variants highlighted in blue correspond to variants flanking the alternatively spliced exon 31 [23a], with the full description of these variants at cDNA level (panel **B**). Each number in the circle corresponds to the total number of individuals heterozygous for a specific variant. The figure was prepared using the ProteinPaint application (Zhou et al. 2016). CSRD (cysteine-serine rich domain) is depicted in green, TBD (tubulin-binding domain) in red, GRD (GAP- related domain) in purple, Sec14 (Sec14 homologous domain) in yellow, PH (pleckstrin homologoy domain) in grey and CTD (C-terminal domain) in blue

Based on the observed effect on splicing, variants were grouped according to their expected effect at the protein level. Twenty-one different NF1 amino acids were affected (being the amino acid where the frameshift leading to a premature stop codon started), with seven specific amino acids most often observed affecting 155/200 (77.5%) cases, i.e. p.Ser1917 (24%, 48/200), p.Ser421 (12.5%, 25/200), p.Val1371 (10%, 20/200), p.Lys297 (9.5%, 19/200), p.Ser465 (8%, 16/200), p.Gln97 (7.5%, 15/200) and p.Ala548 (6%, 12/200) (Fig. 1A and Figure S2). These recurrently affected amino acids represented 44 *different* PVs (64.7%, 44/68), located in nine specific introns, i.e. 3 [3], 8 [6], 9 [7], 11 [9], 12 [10a], 14 [10c], 30 [23.2], 31 [23a] and 39 [30]. In all, 22/57 *NF1* introns (based on isoform type II) harbored one or more deep intronic (likely) PVs. Average size of the affected introns was ~ 9321 bp (versus ~ 4743 bp for all introns). Larger introns (≥ 1000 bp) were more frequently involved (19/22 of deep intronic-affected introns vs. 16/35 of the remaining introns; P = 0.0024 with Fisher’s exact test).

All variants were divided into five categories depending on their effect on splicing according to the classification proposed by Wimmer et al. (2007) (Figure S1). The vast majority of the PVs (56/68) are 177–27,385 bp away from the closest exon–intron boundary and lead to a type II splice effect, i.e. exonization of deep intronic sequences which leads in 54/56 of the variants to an out-of-frame (OOF), in one to an in-frame (IF) and in one both OOF and IF effects. Seven variants located 21–449 bp away from the closest exon–intron boundary generate a novel splice site (2/7 a 3’ and 5/7 a 5’ splice site) that is used instead of the natural splice site (splice variants type III). In all seven cases this leads to exonization of OOF intronic sequences flanking an exon. Another three variants lead to skipping of an IF (2/3) or an OOF (1/3) exon (type I splice effect) and two to use of a pre-existing 3’ splice site (type IV splice effect). The latter five variants are located 21–60 bp upstream of an intron–exon boundary and are assumed to affect regulatory elements such as the branch-point or the AG-exclusion zone at the 3’end of introns (Wimmer et al. 2020). No type V splicing variants have been observed in the studied cohort. In total, 64/68 different deep intronic PVs lead to a premature termination codon (PTC) and, hence, are expected to result in NMD, 3/68 variants cause an IF splice effect and 1/68 causes two different effects, one being an IF and one an OOF effects.

### Identification of nine different pathogenic deep intronic variants flanking exon 31 [23a] in 20 unrelated individuals

We identified five different deep intronic variants in intron 30 [23.2] and four in intron 31 [23a] in a total of 20 unrelated individuals (Fig. 1B), and found they predominantly affected the *NF1* isoform II (Table S1 and Table S3). The aberrantly spliced transcripts caused by these deep intronic variants all result in a PTC (Table 1).

**Table 1:** List of *NF1* (likely) pathogenic deep intronic variants flanking exon 31 [23a], with prediction of 5’ and 3’ splice sites by *in-silico* tools

cDNA variant description	5' splice donor site						3' splice acceptor site						Branch point
	Sequence	SSF-like	MaxEntScan	NNSplice	Splice Finder	SpliceAI Δscore (Donor Gain)	Sequence	SSF-like	MaxEnt Scan	NNSplice	Splice Finder	SpliceAI Δscore (Acceptor Gain)	Alamut
NM_000267.3:c.4110 + 937C > G	*WT* TGAGGCTGCT**C**TATGTGAATT	0.00	0.00	0.00	0.00	0.54	CTCTCTTTGC**AG**AGTCATCGCA	89.75	12.78	0.98	10.82	0.10	NA
	*Mut* TGAGGCTGCT**GT**ATGTGAATT	0.00	5.10	0.00	0.00								
NM_000267.3:c.4110 + 945A > G	*WT* GCTGCTCTATGTGA**A**TTTTTTC	0.00	0.00	0.00	0.00	0.99	CTCTCTTTGC**AG**AGTCATCGCA	89.75	12.78	0.98	10.82	0.14	NA
	*Mut* GCTGCTCTAT**GT**GA**G**TTTTTTC	78.89	5.56	0.92	0.00								
NM_000267.3:c.4110 + 973 T > G	*WT* TTTTAAAGA**T**GTATCTGTTCTA	0.00	3.04	0.00	0.00	0.93	CTCTCTTTGC**AG**AGTCATCGCA	89.75	12.78	0.98	10.82	0.13	NA
	*Mut* TTTTAAAGA**GGT**ATCTGTTCTA	74.08	7.27	0.84	0.00								
NM_000267.3:c.4110 + 2183G > T / NM_001042492.2:c.4111-1636G > T	*WT* ATGTTGAAAGG**G**AAGTGAGTAT	0.00	0.00	0.00	0.00	0.96	TGCCTTTGGCAGTAACAGAAAG	76.21	8.49	0.91	0.86	0.70	NA
	*Mut* ATGTTGAAAG**GT**AAGTGAGTAT	99.69	11.00	1.00	5.67								
NM_000267.3:c.4111-3485 T > G / NM_001042492.2:c.4173 + 1859 T > G	*WT* AATCTAATTGGTAA**T**TCGAGAT	74.97	5.73	0.82	0.00	0.93	TGTATTTCTCAGCACAGGTAAT	77.74	4.48	0.00	0.00	0.82	NA
	*Mut* AATCTAATTG**GT**AA**G**TCGAGAT	87.36	10.47	1.00	0.00								
NM_000267.3:c.4110 + 2424A >G / NM_001042492.2:c.4111-1395A > G	TTCTCGTATG**GT**GAGAGAATTC	81.49	7.23	0.84	0.00	0.43	*WT* TGATGGTTTTAGGAT**A**AAGATA	75.49	9.05	0.58	3.12	0.37	NA
							*Mut* TGATGGTTTT**AG**GAT**G**AAGATA	75.49	9.05	0.81	3.33		
NM_000267.3:c.4110 + 4159A > G / NM_001042492.2:c.4173 + 278A > G	TTGTTTGAAG**GT**AATGTGAGTG	82.28	8.99	0.44	0.00	0.90	*WT* GTTACTTTTTA**A**ATTATAAATG	0.00	0.00	0.00	0.00	0.94	NA
							*Mut* GTTACTTTTT**AG**ATTATAAATG	86.73	10.34	0.98	5.94		
NM_000267.3:c.4110 + 4166A > G / NM_001042492.2:c.4173 + 285A > G	TTGTTTGAAG**GT**AATGTGAGTG	82.28	8.99	0.44	0.00	0.91	*WT* TTTAAATTATA**A**ATGAATGCAA	0.00	0.00	0.00	0.00	0.89	NA
							*Mut* TTTAAATTAT**AG**ATGAATGCAA	84.38	8.08	0.94	6.48		
NM_000267.3:c.4111-679G > A / NM_001042492.2:c.4174-679G > A	TAAATAATAG**GT**AGGTTCTTGT	85.79	7.92	0.97	0.00	0.44	*WT* TTTTTCTTTTAGGTCCCCAAGA *Mut* TTTTTCTTTT**AG**GTCCCCAAGA	93.5293.52	10.4610.46	1.001.00	5.187.25	0.21	096.44
	AACATTACTT**GT**AAGTAAGTTT	78.87	8.00	0.93	0.00	NA							
	TTACTTGTAA**GT**AAGTTTTTTT	83.74	8.07	0.77	0.00	NA							

### In-silico analysis of mis-splicing caused by deep intronic variants flanking exon 31 [23a]

The *in-silico* analysis of the deep intronic variants enhanced our understanding of the mechanisms underlying the splicing defects caused by these variants (Table 1 and Table S4a-d). A total of 20 distinct deep intronic variants reported in the UAB dataset were predicted to either create a de novo canonical donor site or activate a cryptic splice donor site. In addition, 24 of the variants were predicted to create a de novo canonical splice acceptor site or activate a cryptic splice acceptor site. Several variants, e.g. NM_000267.3:c.4110 + 937C > G, NM_000267.3:c.4110 + 945A > G and NM_000267.3:c.4110 + 973 T > G, were predicted to create or activate each a *different* splice donor site, which used the same cryptic splice acceptor site upstream to exonize the intervening intron 30 [23.2] sequence (Figure S3). This specific activated cryptic acceptor site (ctctctttgc**AG**agtcatcgca) had a high predicted splice strength in several predictions programs, i.e. 0 / 78.89 / 74.08 of SSF-like, 5.10 / 5.56 / 7.27 of MaxEntScan, 0 / 0.92 / 0.84 of NNSplice and 0.54 / 0.99 / 0.93 of SpliceAI Δ score (Table 1). Other variants, e.g. NM_000267.3:c.4110 + 2183G > T and NM_001042492.2:c.4173 + 1859 T > G, were predicted to create or activate a good splice donor site that was utilized by the splicing machinery along with cryptic splice acceptor sites (Figure S4), that however had only moderate splice strength according to several prediction programs (Table 1). For NM_00267.3:c.4110 + 2424A > G (Table 1), NNSplice indicated a slight increase of the strength of the splice acceptor site (tgatggtttt**AG**gat**g**aagata). As this variant is four nucleotides downstream of this cryptic splice acceptor site, which is however not accounted for in the consensus sequence for splicing prediction, the variant was not predicted to affect the splice site by MaxEntScan (Table 1). NM_001042492.2: c.4173 + 278A > G (described by Kannu et al. 2013) and NM_001042492.2:c.4173 + 285A > G (Figure S4) were both predicted to create good splice acceptor sites, utilizing the same cryptic splice donor site downstream to exonize the intervening intron 31 [23a] sequence. NM_001042492.2:c.4174-679G > A is located 20 nucleotides upstream of a cryptic splice acceptor site (**g**tgagatccttttttctttt**AG**gtccccaaga) with a good splice strength as predicted by most programs. The variant NM_001042492.2:c.4174-679G > A (**a**tgagatccttttttctttt**AG**gtccccaaga) does not alter the predicted strength of this acceptor site, however the branch point prediction integrated in Alamut Visual Plus™ software v.1.6.1 predicts that c.4174-679G > A creates a strong branch point (score of 96.44). Therefore, *in-silico* analysis predicts that the variant c.4174-679G > A likely causes the observed aberrant splicing by creating a de novo branch point (Table 1).

### NM_001042492.2:c.4110 + 2424A > G results exclusively in mis-splicing of NF1 transcript II

To assess with refined assays whether variant NM_001042492.2:c.4110 + 2424A > G affects both or only one of the *NF1* isoforms, we PCR-amplified NF1 transcripts from the NF1 negative controls and the individual UAB-R5431 carrying this variant. We analyzed the resulting PCR products by agarose gel and the more sensitive method of capillary electrophoresis fragment analysis. Both methods consistently showed that two fragments of different sizes, i.e. 295 bp representing the *NF1* transcript type I excluding exon 31 [23a] and 358 bp representing the transcript type II including exon 31 [23a] were amplified from the NF1-negative controls and three fragments were amplified from the cDNA of the individual UAB-R5431 (Figure S4). These included in addition to the two fragments amplified also in the NF1-negative controls a fragment of 399 bp derived from the NF1 transcript type II containing a 41 bp cryptic intron inserted between exons 30 [23.2] and 31 [23a] (NM_001042492.2:r.4110_4111ins4111-1398_4111-1358).

To further confirm the origins of the splicing patterns resulting from NM_001042492.2:c.4110 + 2424A > G, we cloned the PCR products amplified from the cDNA into TOPO®-TA cloning® vector pCR 4-TOPO. Subsequently, 108 clones were sequenced in both directions. Forty-nine out of 108 clones were derived from the normal *NF1* isoform I and 32 out of 108 clones were from the normal *NF1* isoform II. Twenty-seven out of the 108 clones resulted from the *NF1* isoform II, containing the 41 bp cryptic exon from intron 30 [23.2] (Table S5). No clones containing the 336 bp fragment, deriving from the 295 bp and 41 bp, representing the *NF1* transcript type I and a cryptic exon from intron 30 [23.2], respectively, were detected within 108 clones. Therefore, all results consistently indicated that in UAB-R5431 the NM_001042492.2:c.4110 + 2424A > G variant caused exonization of a 41 bp cryptic exon from intron 30 [23.2], affecting exclusively the *NF1* transcript type II, including the alternatively spliced exon 31 [23a].

### All other deep intronic variants identified in intron 30 [23.2] or 31 [23a] caused mis-splicing mainly affecting the NF1 transcript type II

NM_001042492.2:c.4110 + 937C > G, NM_001042492.2:c.4110 + 945A > G, NM_001042492.2:c.4173 + 285A > G, NM_001042492.2:c.4173 + 278A > G, NM_000267.3:c.4110 + 2183G > T and NM_001042492.2:c.4173 + 1859 T > G, all resulted in mis-splicing by insertion of a cryptic exon mainly in the *NF1* transcripts type II. Results of the capillary gel electrophoresis fragment analysis and cloning followed by sequencing are summarized in Table S5.

### NM_001042492.2:c.4174-679G > A deep intronic variant results in a complex splicing pattern

The NM_001042492.2:c.4174-679G > A variant was identified in six unrelated patients (Table S1). This variant is located 22 nucleotides upstream of a cryptic exon and the variant is predicted to activate a branch site (Table 1).

Besides the fragments derived from the wild type *NF1* transcripts type I (295 bp) and type II (358 bp), minor quantity of fragments of 457 bp and 394 bp were observed by fragment analysis and by sequencing to represent the insertion of a 99 bp cryptic exon into the *NF1* isoform II as well as isoform I (Figure S5). NNSplice showed the presence of a strong cryptic splice acceptor and donor (scores 0.97 and 1.00, respectively) that is used by the splicing machinery to exonize the 99 bp cryptic exon (Table 1). Unexpectedly, an additional three other small peaks, representing fragments of 335 bp, 398 bp and 461 bp were also observed in the PCR products from the cDNA amplification. NNSplice predicted presence of an additional cryptic donor site at c.4111-617_4111-616 (taaataatagGTaggttcttgt; score of 0.93) and a third splice donor site at c.4174-554_4174-553 (ttacttgtaaGTaagttttttt; score of 0.77) (Table 1).

We cloned the PCR products into TOPO®-TA cloning® vector pCR 4-TOPO and picked 93 clones from the UAB-R2603 (mosaic case), 108 clones from the UAB-R7963 and 103 clones from the UAB-R4034, and sequenced them in both directions. Sequencing results showed that the 335 bp and a fraction of the 398 bp fragments represented an additional 40 bp cryptic exon in the *NF1* isoform I (r. 4110_4111ins4111-657_4111-618) as well as isoform II (r.4110_4111ins4111-657_4111-559), respectively, in which the cryptic splice donor site c.4111-617_4111-616 was used instead of c.4111-558_4111-557 (Table S6 and Figure S5). Furthermore, another fraction of the 398 bp products represented a 103 bp cryptic exon (c.4110_4111ins4111-657_4111_555) inserted in the *NF1* transcript I, in which the splicing machinery used a different cryptic splice donor site c.4111-554_4111-553 to exonize these 103 bp of intron 31 [23a]. Finally, the 461 bp products were derived from the 103 bp cryptic exon mis-spliced into the *NF1* transcript II.

In all individuals carrying NM_001042492.2:c.4174-679G > A, equal amounts of transcripts I and II containing the 99 bp cryptic exon were observed. The 40 bp and 103 bp cryptic exons were also found in both transcripts I and II, but in a lower fraction of the transcripts compared to the 99 bp cryptic exon. As the individual UAB-R2603 was mosaic (carried the variant with VAF of ~ 35% in blood), cDNA fragment analysis showed a lower peak for all cryptic mis-spliced exons compared to individuals UAB-R7963 and UAB-R4034, in line with mosaicism (Table S6 and Figure S5).

### Deep intronic variants of uncertain significance reported in the literature, but not found in the UAB dataset

A total of 65 different deep intronic (likely) PVs or variants of uncertain significance (VUS) located at ≥ 20 nucleotides from the exon borders (Table S2), were found in the publicly available databases and literature (as of 16^th^ of May, 2022). Among these, 28 were also identified in the UAB cohort and classified as (likely) pathogenic. Four additional variants, NM_000267.3:c.654 + 28A > G, NM_000267.3:c.889-25_889-21del, NM_000267.3:c.1722-24A > G and NM_000267.3:c.4269 + 22_4269 + 25del, were also observed in the UAB dataset, however, as two of these individuals also carried an additional clearly *NF1* PVs or no splicing effect was observed through RNA-based verification (see details in Table S2), these variants should be re-classified as likely benign and/or VUS (not pathogenic) according to the current recommendations (Richards et al. 2015). The remaining 33 variants described in the literature and/or in publicly available databases have never been observed in 8,090 unrelated *NF1* PVs-positive individuals from the UAB dataset molecularly confirmed through comprehensive RNA-based analysis. Among these, based on evidence provided by the reporting authors, only 7/33 variants should be classified as pathogenic or likely pathogenic in line with the current recommendations (see details in Table S2).

### Demographic and clinical characterization of the studied cohort

Detailed demographic and clinical descriptions of the studied cohort are presented in Table 2 and Table S7. Briefly, a total of 200 unrelated individuals carrying one of 68 different *NF1* deep intronic (likely) PVs were enrolled in the study, including 59/200 (29.5%) familial and 85/200 (42.5%) sporadic case subjects; 56/200 (28%) individuals had an unknown family history. The presence of ≥ 6 café-au-lait macules (CALMs) and freckling was observed in 160/185 (86.5%) and 108/164 (65.9%) individuals, respectively, among which 60/78 (76.9%) cases ≥ 9 years old presented with both pigmentary manifestations. Lisch nodules were reported in 33/105 (31.4%) of the studied cohort, but in 27/54 (50%) of individuals ≥ 9 years old. A plexiform neurofibroma was reported in 21/67 individuals ≥ 9 years (31.3%), while ≥ 2 cutaneous and/or subcutaneous neurofibromas were observed in 36/47 (76.6%) NF1-affected adults. A total of 22/146 (15.1%) of the studied cohort developed spinal neurofibromas, including 15/44 (34.1%) individuals ≥ 19 years old. None of the individuals < 5 years old had symptomatic optic pathway gliomas (OPGs) (0/59), but asymptomatic OPGs were present in 4/9 (44.4%) additional children in this age group who underwent MRI examination. Among the individuals ≥ 5 years old, the prevalence of symptomatic and asymptomatic OPGs was 10.5% (10/95) and 14.7% (5/34), respectively. Skeletal abnormalities were reported in 18.2% of the studied cohort (29/159), with scoliosis being the most frequently observed (16/159 all ages, but 8/49 ≥ 19 years). Cognitive impairment and/or learning disabilities affected 45/151 individuals.

**Table 2:** Summary of clinical features of 200 unrelated individuals from the UAB dataset carrying a constitutional *NF1* deep intronic (likely) pathogenic variant

NF1 feature	N (%)						
	0–24 months	2–4 years	5–8 years	9–13 years	14–18 years	≥ 19 years	Total
≥ 6 CALMs	38/42 (90.5)	23/24 (95.8)	23/26 (88.5)	18/18 (100)	16/17 (94.1)	42/58 (72.4)	160/185 (86.5)
Skinfold freckling	9/41 (22)	18/24 (75)	14/21 (66.7)	13/16 (81.3)	13/14 (92.9)	41/48 (85.4)	108/164 (65.9)
Lisch nodules	1/22 (4.6)	1/16 (6.3)	4/13 (30.8)	2/8 (25)	4/8 (50)	21/38 (55.3)	33/105 (31.4)
Major external plexiform neurofibromas	0/39 (0)	1/21 (4.8)	4/24 (16.7)	3/11 (27.3)	3/12 (25)	15/44 (34.1)	26/151 (17.2)
Cutaneous neurofibromas ^A^	0/39 (0)	1/21 (4.8)	3/22 (13.6)	3/15 (20)	3/13 (23.1)	32/51 (62.8)	42/161 (26.1)
Subcutaneous neurofibromas ^A^	0/32 (0)	0/16 (0)	2/12 (16.7)	0/7 (0)	0/7 (0)	14/32 (43.8)	16/106 (15.1)
Symptomatic spinal neurofibromas	0/36 (0)	0/23 (0)	1/19 (5.3)	0/12 (0)	2/12 (16.7)	10/44 (22.7)	13/146 (8.9)
Symptomatic OPGs ^B^	0/36 (0)	0/23 (0)	2/21 (9.5)	1/13 (7.7)	1/13 (7.7)	6/48 (12.5)	10/154 (6.5)
Asymptomatic OPGs ^C^	1/5 (20)	3/4 (75)	1/4 (25)	0/4 (0)	0/5 (20)	4/21 (19.1)	9/43 (20.9)
Skeletal abnormalities	5/37 (13.5)	3/23 (13.0)	3/23 (13.0)	4/14 (28.6)	4/13 (30.8)	10/49 (20.4)	29/159 (18.2)
Cognitive impairment and/or learning disabilities	5/35 (17.1)	6/21 (28.6)	12/23 (52.2)	10/14 (71.4)	4/13 (30.8)	8/45 (17.8)	45/151 (29.8)

^A^ At least two cutaneous / subcutaneous neurofibromas were required to be considered as “positive for the criterion of neurofibromas”. ^B^ The absence of symptomatic OPGs was determined by ophthalmological examination and/or by MRI. ^C^ Including only individuals without signs of symptomatic OPGs who underwent MRI examination

Altogether, 139/184 (75.5%) individuals fulfilled the NIH diagnostic criteria (NIH, 1988), with 125/184 (67.9%) if family history was excluded as criterion. For 16 individuals the phenotypic data was not provided. Among 45/184 (24.5%) who did not fulfill the NIH diagnostic criteria (with 34/45 being ≤ 8 years), 30/45 had only ≥ 6 CALMs, 4/45 presented with spinal neurofibromas-only, while two additional individuals were found to have exclusively ≥ 2 cutaneous neurofibromas or Lisch nodules (2/45). In 28 out of these 36 individuals who had only a single NF1-related clinical sign (77.8%) a clearly pathogenic *NF1* deep intronic variant was identified (details in Table S8). The remaining eight individuals (with 5/8 ≤ 8 years) presented exclusively with ≥ 6 CALMs (2/8) carried a likely pathogenic *NF1* deep intronic variant.

## Discussion

Since the identification of the *NF1* gene and its encoded protein product, neurofibromin, in the 1990s (Ballester et al. 1990; Wallace et al. 1990; DeClue et al. 1991), molecular methods have been developed intensively to identify genetic variants in individuals with a clinical suspicion of NF1. With the comprehensive testing strategy, including direct cDNA sequencing of the entire coding sequence and copy number analysis, the *NF1* PVs are found in about 95% of unrelated individuals with a classical NF1 phenotype (Messiaen et al. 2000, 2009). To date, no large-scale study describing the spectrum of *NF1* deep intronic variants and, more importantly, their well-characterized effect on mRNA splicing, was reported.

Here, we investigated all *NF1* deep intronic (likely) PVs reported in the UAB dataset, consisting of 8,090 unrelated individuals with molecularly confirmed NF1. Notably, all these individuals were tested with an RNA-based approach, providing a functional test to identify all variants affecting splicing. Over fifteen years of such clinical diagnostic testing resulted in the identification of 68 different deep intronic (likely) PVs in 200/8,090 probands, resulting in the overall prevalence of 2.5%. A similar frequency has been observed in the French cohort, in which 13 deep intronic variants out of 546 (2.4%) were found (Sabbagh et al. 2013).

In total, 37/68 deep intronic variants reported in the UAB dataset were classified as *clearly* pathogenic. The remaining 31 variants should be classified as likely pathogenic in line with the current recommendations (Richards et al. 2015) until more evidence becomes available (details in Table S9). Among these, we identified two variants, i.e. NM_000267.3:c.889-21C > A and NM_00267.3:c.1062 + 60A > G, in two unrelated cases (UAB-R0822 and UAB-R9497, respectively) resulting only in a partial skipping of the entire exon 9 [7] (r.889_1062del), 30% and 20%, respectively. Both individuals, being ≤ 14 years old, presented only with multiple typical CALMs, with no further NF1 clinical signs observed (details in Table S7). UAB-R9497’s mother who was not reported to have any NF1 clinical symptoms tested positive for the presence of this specific *NF1* variant. It is possible that this and other variants with a leaky splice effect are hypomorphic and that not all carriers of such variant(s) present with detectable clinical signs of NF1. In a similar case of 34-year-old woman with a leaky *NF1* deep intronic splice variant, NM_000267.3:c.3198-314G > A, the very mild NF1 phenotype was in addition attributed to mosaicism for this PV (Fernandez-Rodriguez et al. 2011), which might be an additional explanation in the case of the UAB-R9497’s mother. Furthermore, it cannot be excluded that the fraction of mis-spliced transcripts varies among different individuals. Unfortunately, suitable material from the mother was not available to test for these possible explanations in this case, and future studies are needed to gain a better understanding of this observation.

To date, 65 distinct deep intronic variants reported by the authors as VUS, likely pathogenic or pathogenic have been described in the literature and/or publicly available databases, i.e. HGMD, LOVD and ClinVar (Table S2). Among these, 28/65 were also observed in the UAB dataset and classified as (likely) pathogenic as NM_000267.3:c.1722-24A > G was reported as VUS in both ClinVar and UAB datasets. NM_000267.3:c.1722-24A > G was observed once in the UAB cohort through DNA-based NGS testing, followed by RNA verification of potential effect on splicing, but no mis-splicing was observed. Therefore, current classification should be VUS until more evidence becomes available. Three another variants, i.e. NM_000267.3:c.654 + 28A > G, NM_000267.3:c.889-25_889-21del, NM_000267.3:c.4269 + 22_4269 + 25del, have been reported as likely benign in the UAB dataset, as the affected individuals carried an additional clearly pathogenic *NF1* variant (details Table S2). We carefully examined the remaining 33 variants, not yet observed in the UAB dataset, for the available evidence in line with the current recommendations and the re-classification for 18 of them is needed (Richards et al. 2015). Briefly, 5/18 deep intronic variants, i.e. NM_000267.3:c.61-16301G > A, NM_000267.3:c.4110 + 3815G > A, NM_000267.3:c.4174-22A > G, NM_000267.3:c.4269 + 22_4269 + 25del and NM_000267.3:c.8051-70A > T, with VUS or conflicting interpretation of classification should be classified as likely benign due to a high prevalence (minor allele frequency > 0.01) across control populations in the Genome Aggregation Database (gnomAD) and based on the normal results of *in-silico* predictions analysis (details in Table S2). Another 13 deep intronic variants were initially classified as pathogenic or likely pathogenic by the original authors despite the paucity of clear evidence, for instance only based on *in-silico* predictions with no RNA verification effect on splicing. As we found several errors in variant nomenclature at both cDNA and gDNA levels, we propose to call these variants still VUS until these discrepancies will be resolved and/or RNA verification on the effect on splicing will be confirmed (details in Table S2).

Taken together, these data clearly demonstrated the difficulties associated with proper interpretation of the pathogenicity of deep intronic variants, especially those not previously described. With the significant development of new molecular technologies, the current diagnostic challenge involves not only the cost-effectiveness and sensitivity of available genetic testing techniques, but also variants’ pathogenicity evaluation. Proper classification is the first step to correctly define the cause of the genetic disorder and further properly evaluate the implications for reproductive risk. For genetic disorders such as NF1, with wide *NF1* allelic heterogeneity and a substantial fraction of splicing variants, gDNA sequencing assays alone are not enough to 1/ detect deep intronic variants affecting splicing and 2/ decide on the pathogenicity of missense or even “silent” variants identified by gDNA analysis if not previously reported. Of note, *in-silico* splicing prediction tools work well for variants located adjacent to exon boundaries and not further than two nucleotides from the exon border. However, for variants located outside of the canonically conserved splice sites, the specificity of cryptic or novel splice sites predictions is limited, similar as predictions for the effect of variants on splice enhancer / silencer sites or potential branch points (Taggart et al. 2012). Therefore, a new variant should be subjected to extensive RNA analysis to assess functionally the effect on splicing in patient-derived samples (containing the entire patient-specific sequence, as opposed to the limited sequence content available in minigene experiments) as was performed in the current study.

The *NF1* gene consists of 57 constitutive exons and three well-validated alternatively spliced exons, including exon 31 [23a]. The alternatively spliced exon 31 [23a] is a 63 bp in-frame cassette exon. Based on its presence or absence two NF1 isoforms are distinguished, i.e. type I (without exon 31[23a]) and type II (with the additional 21 amino acids from exon 31 [23a], when translated). In this study, we identified nine distinct deep intronic variants flanking the alternatively spliced exon 31 [23a] in 20 unrelated individuals (Fig. 1B, Table 1 and Table S1). Through comprehensive molecular analysis, we showed that these variants caused mis-splicing preferentially including exon 31 [23a] in the transcript (Figure S3-S5). As exon 31 [23a] is located in the GRD domain, the question whether these additional amino acids affect somehow the Ras-GTP activity of neurofibromin was raised. Since many years it has been widely known that the isoform type II has several times lower Ras-GTP activity than isoform type I and exon 31 [23a] is predominantly skipped specifically in human central nervous system (Uchida et al. 1992; Andersen et al. 1993). However, the biology of this alternative splicing event is still unclear. Here, we demonstrated that despite the reported lower Ras-GTP activity of the isoform type II, individuals carrying these specific variants still presented with a classical NF1 phenotype. Indeed, among 9/20 affected probands being ≥ 14 years old, 7/9 fulfilled the NIH diagnostic criteria based on the presence of multiple cutaneous manifestations, Lisch nodules and/or different types of neurofibromas (Table S7); for UAB-R0338 the phenotypic information was not available. A single individual (UAB-R6201) ≥ 19 years old, carrying a heterozygous NM_000267.3:c.4110 + 937C > G, presented with symptomatic spinal neurofibroma(s) and < 6 CALMs only, therefore not fulfilling the former NIH diagnostic criteria. However, NM_000267.3:c.4110 + 937C > G was found in two additional unrelated individuals from the UAB dataset (UAB-R01511FN.204 and UAB-R59921FN.205), identified through DNA-based NGS testing, followed by the RNA-based confirmation of mis-splicing. Both subjects, although younger ≤ 14 years old, had ≥ 6 CALMs in both cases and faint bilateral axillary freckling in one of them (details in Table S7). Therefore, as the UAB-R6201 subject carried a *clearly* pathogenic *NF1* variant with a variant allele fraction of ~ 50% in peripheral blood lymphocytes, this individual eventually fulfilled the NF1 diagnostic criteria in line with the recently updated recommendations (Legius et al. 2021).

The RNA-based molecular approach used in our institution allowed to confirm the NF1 diagnosis in an additional 28 unrelated individuals (with 24/28 being ≤ 14 years old) who presented exclusively with a single NF1 clinical sign at the time of genetic testing (Table S8). This data clearly showed the importance of performing molecular analysis of the most frequently affected deep intronic *NF1* regions during the routine *NF1* clinical genetic testing.

Mouse models are extremely useful to understand the biology of disease-associated phenotypes. To test the biological importance of exon 31 [23a] inclusion, Costa et al. (2001) generated a mouse model with biallelic knock out of the exon 31 [23a]. Learning and memory tests between wild-type and mutant mice showed clear impairments in cognitive functioning in mutant mice, suggesting a critical role of alternative splicing of exon 31 [23a] for brain function. In line with these findings, Assunto et al. (2019) confirmed the association between the lower levels of the NF1 isoform type I and the occurrence of learning disability/intellectual disability in NF1-affected individuals. In the current study, no statistical difference for prevalence of cognitive impairment between individuals carrying the *NF1* deep intronic PVs flanking exon 31[23] and the remaining NF1-affected individuals was observed (5/17 vs. 40/134, *P* = 1.0 with Fisher’s exact test).

## Conclusion

This study provides a state-of-the-art description and summary of currently known *NF1* deep intronic (likely) PVs with well-documented effect on splicing. A multi-step approach using an RNA-based core assay, complemented with additional gDNA-based methods, allowed for a detailed identification and characterization of such variants in the UAB dataset. We also curated all available records of deep intronic variants classified as VUS, likely pathogenic, pathogenic or with conflicting interpretations from the literature and/or publicly available databases (as of 16th of May, 2022) in line with the current diagnostic recommendations (Richards et al. 2015). Of note, our data demonstrate that loss-of-function *NF1* variants specifically affecting isoform type II, at least as evidenced by studying RNA from lymphocytes of NF1-affected individuals, still can result in a classic NF1 phenotype. We believe that the observed effect of the herein reported deep intronic variants flanking exon 31 [23a] might allow further research into the alternative splicing and the DNA sequences essential to this process. The unique list of 75 *NF1* deep intronic (likely) PVs, including 68 variants from the UAB dataset and additional seven from the literature (Table S9), is of immediate utility to clinical diagnostic laboratories conducting *NF1* clinical genetic testing and these variants should be implemented in the testing approaches.

## Supplementary Information

Below is the link to the electronic supplementary material.

Supplementary file1 (PDF 2454 KB)

Supplementary file2 (XLSX 16643 KB)

## Data Availability

All deep intronic (likely) pathogenic variants in the *NF1* gene described in this study were submitted to the LOVD and ClinVar databases.

## Declarations

### Web resources

ClinVar, https://www.ncbi.nlm.nih.gov/clinvar/

GeneReviews, Friedman, J.M. (2014). Neurofibromatosis 1, http://www.ncbi.nlm.nih.gov/books/NBK1109

gnomAD, https://gnomad.broadinstitute.org/

GraphPad, https://www.graphpad.com/quickcalcs/

HGMD, http://www.hgmd.cf.ac.uk/ac/index.php

HGVS, http://varnomen.hgvs.org

LOVD, https://databases.lovd.nl/shared/genes/NF1

MaxEntScan, https://www.genes.mit.edu/burgelab/maxent/Xmaxentscan_scoreseq.html

NNSplice, http://www.fruitfly.org/seq_tools/splice.html

OMIM, http://www.omim.org/

ProteinPaint, https://proteinpaint.stjude.org/

SpliceAl, https://spliceailookup.broadinstitute.org/

SpliceSiteFinder-like, http://www.genet.sickkids.on.ca/~ali/splicesitefinder.html.
