# Supplementary material for: Analysis of 200 unrelated individuals with a constitutional NF1 deep intronic pathogenic variant reveals that variants flanking the alternatively spliced NF1 exon 31 [23a] cause a classical neurofibromatosis type 1 phenotype while altering predominantly NF1 isoform type II
Source: Hum Genet. 2023 Apr 25;142(7):849–61. doi: 10.1007/s00439-023-02555-z (PMC10329576; doi:10.1007/s00439-023-02555-z)
Supplement: Supplementary file 1 — Supplementary file1 (PDF 2454 KB) [file 439_2023_2555_MOESM1_ESM.pdf]

SUPPLEMENTARY INFORMATION

Analysis of 200 unrelated individuals with a constitutional *NF1* deep intronic pathogenic variant reveals that variants flanking the alternatively spliced *NF1* exon 31 [23a] cause a classical neurofibromatosis type 1 phenotype while altering predominantly *NF1* isoform type II

Magdalena Koczkowska,[1,2,#] Yunjia Chen,[1,#] Jing Xie,[1,#,*] Tom Callens,[1] Alicia Gomes,[1] Katharina Wimmer,[3] Ludwine M. Messiaen [1]

[1] Department of Genetics, University of Alabama at Birmingham, Birmingham, AL 35294, USA;

[2] 3P-Medicine Laboratory, Medical University of Gdansk, Gdansk, 80-211, Poland;

[3] Institute of Human Genetics, Medical University of Innsbruck, Innsbruck, 6020, Austria;

[#] Drs. Koczkowska, Chen and Xie, contributed equally to this work;

[*] Current affiliation as follows: Natera, Inc., San Carlos, California, USA.

Corresponding author

Ludwine M. Messiaen, Medical Genomics Laboratory, Department of Genetics, University of Alabama at Birmingham, AL 35294, USA, phone: 205.934.5562; email: lmessiaen@uabmc.edu

---

Table S1. Detailed list of (likely) pathogenic deep intronic variants in the *NF1* gene reported in the UAB cohort of 8,090 *NF1* pathogenic variant-positive unrelated individuals, all molecularly confirmed through RNA-based comprehensive analysis.

Table S2. List of deep intronic variants in the *NF1* gene reported in the literature and/or publicly available databases (as of 16th of May, 2022).

Table S3. List of primers used for identification of the *NF1* deep intronic variants flanking exon 31 [23a] at genomic DNA level.

Table S4a. Prediction of 5' and 3' splice sites by *in-silico* tools of the *NF1* deep intronic variants reported in the UAB dataset (deep intronic variants that lead to an intronic sequence being "exonized" with the novel [or enhanced cryptic] splice acceptor/donor and a cryptic splice donor/acceptor).

Table S4b. Prediction of 5' and 3' splice sites by *in-silico* tools of the *NF1* deep intronic variants reported in the UAB dataset (deep intronic variants that lead to replacement of the canonical splice donor/acceptor with a novel splice donor/acceptor or splice donor/acceptor with predicted increased strength).

Table S4c. Prediction of 5' and 3' splice sites by *in-silico* tools of the *NF1* deep intronic variants reported in the UAB dataset (deep intronic variants that lead to skipping of an exon with or without creating a novel splicing site).

Table S4d. Prediction of 5' and 3' splice sites by *in-silico* tools of the *NF1* deep intronic variants reported in the UAB dataset (a deep intronic variant that leads to *two* non-continuous intronic sequences being "exonized" with the novel [or *in-silico* predicted increased-strength cryptic] splice acceptor/donor and a cryptic splice donor/acceptor).

Table S5. Results of fragment analysis and cloning of the *NF1* deep intronic variants flanking exon 31 [23a].

Table S6. Results of fragment analysis and cloning of the *NF1* deep intronic c.4714-679G>A variants.

Table S7. Clinical details for 200 unrelated individuals from the UAB cohort carrying one of the *NF1* deep intronic (likely) pathogenic variants.

Table S8. List of 36 individuals from the UAB dataset presented with a single NF1-related clinical sign and carried a (likely) pathogenic *NF1* deep intronic variant, allowing for making the NF1 diagnosis in line with the updated diagnostic criteria (Legius et al. 2021).

Table S9. The unique list of 75 (likely) pathogenic deep intronic variants in the *NF1* gene reported in the UAB dataset and/or HGMD/LOVD/ClinVar databases (as of 16th of May, 2022), with the pathogenicity evaluation according to the current recommendations (Richards et al. 2015).

Figure S1. Schematic representation by Abramowicz & Gos (2018) of five variant categories depending on their effect on splicing according to the classification proposed by Wimmer et al. (2007).

Figure S2. Spectrum of the most recurrent *NF1* deep intronic variants identified in the studied cohort of 200 unrelated individuals from the UAB dataset.

Figure S3. The splicing patterns caused by the *NF1* c.4110+937C>G, c.4110+945A>G and c.4110+973T>G deep intronic variants.

Figure S4. Characterization of the selected *NF1* deep intronic variants and its effect on the splicing patterns.

Figure S5. The complex splicing patterns caused by c.4174-679G>A.

Table S1. Detailed list of (likely) pathogenic deep intronic variants in the *NF1* gene reported in the UAB cohort of 8,090 *NF1* pathogenic variant-positive unrelated individuals, all molecularly confirmed through RNA-based comprehensive analysis.

Table S1 is included as a separate Excel file.

[1] The sign "+" means that the common wild-type sequence of splice acceptor and/or donor sites was used by different variants, shown in their separate boxes, columns F and G (details in Table S4). [2] Importantly, this variant reported in a single individual from the UAB dataset was deposited previously in the HGMD and ClinVar databases (details are provided in Table S2), meaning that this variant described in the current paper and in these databases belongs to the same individual.

Abbreviations: HGMD - Human Genome Mutation Database; LOVD - Leiden Open Variant Database; OOF - out-of-frame; IF - in-frame.

Table S2. List of deep intronic variants in the *NF1* gene reported in the literature and/or publicly available databases (as of 16th of May, 2022).

Table S2 is included as a separate Excel file.

[1] List of all references is provided in the Supplemental Data. [2] The variants descriptions are shown here as reported by the original author(s). [3] The number of unrelated individuals carrying the *NF1* deep intronic variant is provided in round brackets. [4] The classification of the variants deposited in the publicly available databases (LOVD, ClinVar and HGMD) are shown here as reported by the original author(s). Additionally, the evidence originally provided for those variants were reviewed and classification was re-classified by the authors of this study, if needed, based on the ACMG/AMP criteria for pathogenicity (Richards et al. 2015). Details are available in Table S9.

Abbreviations: DM - disease-causing; DM? - likely disease-causing; NA - not applicable; HGMD - Human Genome Mutation Database; LOVD - Leiden Open Variant Database; OOF - out-of-frame; IF - in-frame; NGS - next-generation sequencing; NMD - nonsense mediated decay; HGVS - Human Genome Variant Society; WT - wild type.

Table S3. List of primers used for identification of the *NF1* deep intronic variants flanking exon 31 [23a] at genomic DNA level.

Table S3 is included as a separate Excel file.

Table S4. Prediction of 5' and 3' splice sites by *in-silico* tools of the *NF1* deep intronic variants reported in the UAB dataset:
4a - deep intronic variants that lead to an intronic sequence being "exonized" with the novel [or enhanced cryptic] splice acceptor/donor and a cryptic splice donor/acceptor).
4b - deep intronic variants that lead to replacement of the canonical splice donor/acceptor with a novel splice donor/acceptor or splice donor/acceptor with predicted increased strength).
4c - deep intronic variants that lead to skipping of an exon with or without creating a novel splicing site).
4d - a deep intronic variant that leads to *two* non-continuous intronic sequences being "exonized" with the novel [or *in-silico* predicted increased-strength cryptic] splice acceptor/donor and a cryptic splice donor/acceptor).

Table S4 is included as a separate Excel file.

Table S5. Results of fragment analysis and cloning of the *NF1* deep intronic variants flanking exon 31 [23a].

Table S5 is included as a separate Excel file.

[1] The peak is present and absent when described with "+" and "-", respectively. The peak is present, but at a very low level, when described as "Very Low".

Abbreviations: F - Fragment analysis; TA - Cloning.

Table S6. Results of fragment analysis and cloning of the *NF1* deep intronic c.4174-679G>A variants.

Table S6 is included as a separate Excel file.

[1] The peak is present and absent when described with "+" and "-", respectively. The peak is present, but at a very low level, when described as "Very Low".

Abbreviations: F - Fragment analysis; TA - Cloning.

Table S7. Clinical details for 200 unrelated individuals from the UAB cohort carrying one of the *NF1* deep intronic (likely) pathogenic variants.

Table S7 is included as a separate Excel file.

Abbreviations: F - female; M - male; NS - not specified; i.e. no value provided on the phenotypic checklist; UN - unknown; CALMs - café-au-lait macules; bil - bilateral; gr - groin; ax - axillary; OPG - optic pathway glioma; HTL - hypertelorism; MH - midface hypoplasia; LPH - low posterior hairline; SHWN - short webbed neck; SS - short stature: ADD - attention deficit disorder; ADHD - attention deficit hyperactivity disorder; LD - learning disability; SD - speech delay; MRI - magnetic resonance imaging; JMML - juvenile chronic myeloid leukemia; NGS - next-generations sequencing.

[1] For the variants highlighted with # the effect at the RNA and protein levels has been reported as minor with the *NF1* NM_00267.3 transcript (see details in Supp. Table S1 for the NM_001042492.2 *NF1* transcript effect). [2] A total of 31 unrelated individuals were proven to have a *de novo* variant, with both parents not carrying this particular variant in the blood as shown by testing, however, the formal confirmation of paternity/maternity by identity testing was not performed.

Table S8. List of 36 individuals from the UAB dataset presented with a single NF1-related clinical sign and carried a (likely) pathogenic *NF1* deep intronic variant, allowing for making the NF1 diagnosis in line with the updated diagnostic criteria (Legius et al. 2021).

Table S8 is included as a separate Excel file.

[1] For the variants highlighted with # the effect at the RNA and protein levels has been reported as minor with the *NF1* NM_00267.3 transcript (see details in Table S1 for the NM_001042492.2 *NF1* transcript effect). [2] YES - this individual carries the *NF1* deep intronic variant that is classified as **pathogenic** in line with the current recommendations (details in Table S9); NO – this individual carries the *NF1* deep intronic variant that is classified as **likely pathogenic** in line with the current recommendations (details in Table S9).

Table S9. The unique list of 75 (likely) pathogenic deep intronic variants in the *NF1* gene reported in the UAB dataset and/or HGMD/LOVD/ClinVar databases (as of 16th of May, 2022), with the pathogenicity evaluation according to the current recommendations (Richards et al. 2015).

Table S9 is included as a separate Excel file.

[1] The PS3 criterion has been fulfilled if a deleterious effect on splicing was confirmed by RNA-based study. [2] The PS4 criterion has been fulfilled when the variant was observed in at least two unrelated individuals. [3] The variant c.1527+1159C>T has been reported once in the UAB dataset in a single individual (details in Table S7), but at least two additional cases carrying this variant have been described in the publicly available databases and/or literature. [4] The variant c.4515-21T>C has been reported twice in the UAB dataset, i.e. in a single individual that was included in the cohort described here (UAB-R8343) and in the additional unrelated individual tested with the NGS-based approach, with further RNA-based confirmation of deleterious effect on splicing (details in Table S7). [5] Although the variant c.6579+26C>G has been reported only once in the UAB dataset, this variant with the same deleterious effect on splicing confirmed through RNA-based approach has been described several times in the publicly available databases (details in Table S2). [6] These variants have been originally reported by Sabbagh et al. (2013), but also deposited in the LOVD database (the same reference is present in LOVD), therefore, they should be counted with caution to avoid the repetition of the same individual into calculations. [7] These variants, i.e. c.889-21C>A and c.4110+937C>G, have been reported once in the gnomAD database. [8] The variant c.4515-21T>G is a novel change at the same residue where a different change determined to be pathogenic has been seen before (see details for the c.4515-21T>C in the Table S7). [9] Details of co-segregation with disease in affected family members and phenotypic information are available in Table S7. [10] The proband's mother presenting with no clinical signs of NF1 was tested positive for the *NF1* c.1062+60A>G (details in Table S7). [11] The origin of the variant c.1527+1167C>G was familial, however no further phenotypic details were available. [12] The presence in the following publicly available databases has been verified, i.e. LOVD, ClinVar and HGMD, with all details provided in Table S1 and Table S2. [13] This individual had only ≥6 CALMs, but *SPRED1* genetic testing was negative, thus Legius syndrome can be likely excluded; however, the proband's mother who was reported without any clinical signs of NF1 was tested positive for this specific *NF1* variant, therefore we decided not to include PP4 as fulfilled for this specific variant. [14] As these individuals were reported to have only multiple CALMs and/or skin freckling and no *SPRED1* genetic testing was performed, we decided to not include PP4 as fulfilled for these specific variants.

Abbreviation: PVS1 - null variant (nonsense, frameshift, canonical +/- 1 or 2 splice sites, initiation codon, single or multiexon deletion) in a gene where LOF is a known mechanism of disease; PS1 - same amino acid change as a previously established pathogenic variant regardless of nucleotide change; PS2 - de novo (both maternity and paternity confirmed) in an individual with the disease and no family history; PS3 - well-established in vitro or in vivo functional studies supportive of a damaging effect on the gene or gene product; PS4 - the prevalence of the variant in affected individuals is significantly increased compared to the prevalence in controls; PM1 - located in a mutational hot spot and/or in critical functional domain; PM2 - absent from controls (or at extremely low frequency if recessive) in Exome Sequencing Project, 1000 Genomes, or Exome Aggregation Consortium; PM3 - for recessive disorders, detected in trans with a pathogenic variant; PM4 - protein length changes due to in-frame or stop-loss variants; PM5 - novel missense change at amino acid residue where a different pathogenic missense change has been seen before; PM6 - assumed de novo, but without confirmation of paternity and maternity; PP1 - co-segregation with disease in multiple affected family members; PP2 - missense variant in a gene that has a low rate of benign missense variation; PP3 - multiple lines of computational evidence support a deleterious effect on the gene or gene product (conservation, evolutionary, splicing impact); PP4 - individual's phenotype or family history is highly specific for a disease with a single genetic etiology; PP5 - reputable source reports variant as pathogenic; N/A - not applicable; YES - the criterion has been fulfilled; NO: the criterion has not been fulfilled; ? - due to the lack of evidence, it is difficult to establish whether the criterion has been fulfilled or not; LOF - loss-of-function; ND - no data available.

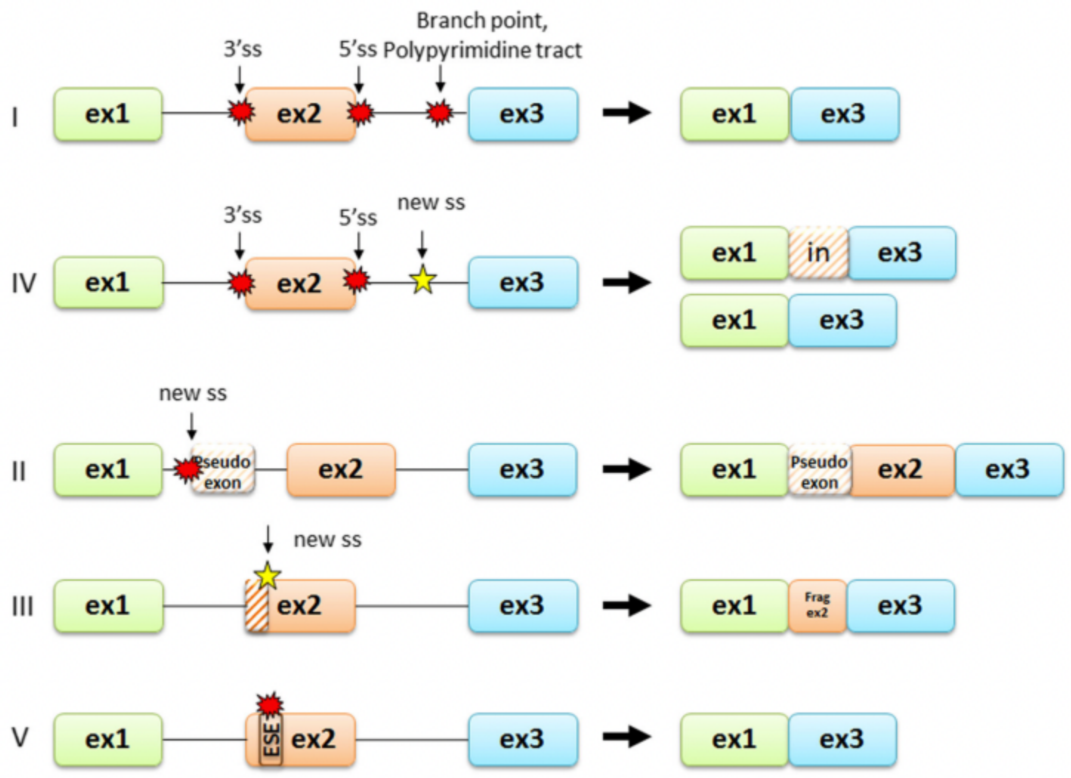

Figure S1. Schematic representation by Abramowicz & Gos (2018) of five variant categories depending on their effect on splicing according to the classification proposed by Wimmer et al. (2007).

Briefly, categories I-V describe the following variants: variants within the canonical splice sites that lead to exon skipping (I), deep intronic variants causing pseudoexon inclusion (II), variants creating *de novo* splice sites that lead to loss of an exon fragment (III), variants in the canonical splice sites that lead to cryptic splice site activation (IV) and variants within exons causing exon skipping (V) (adapted from Wimmer et al. 2007).

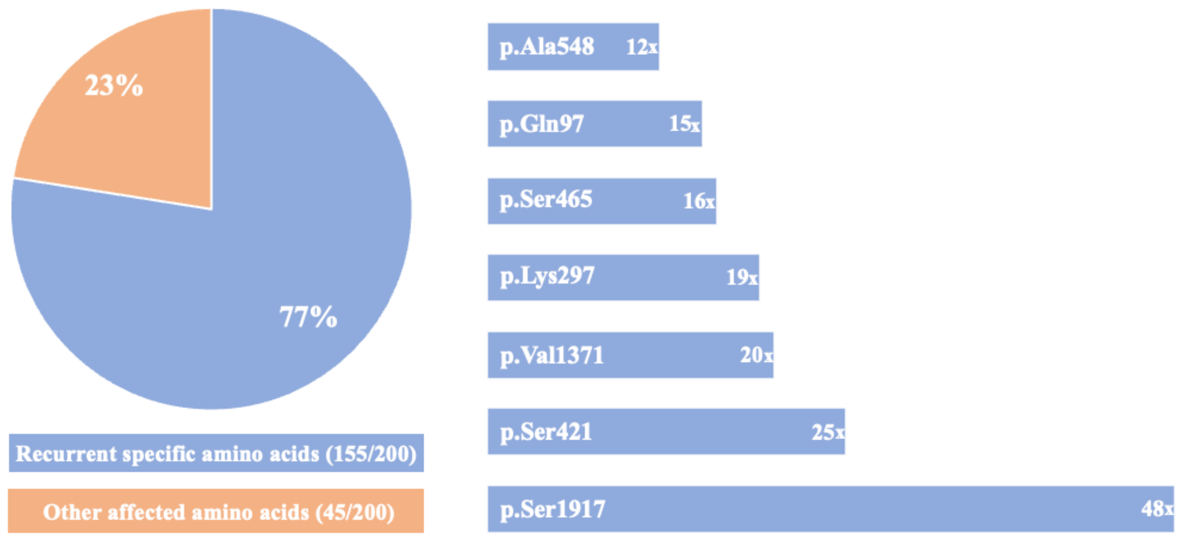

Figure S2. Spectrum of the most recurrent *NF1* deep intronic variants identified in the studied cohort of 200 unrelated individuals from the UAB dataset.

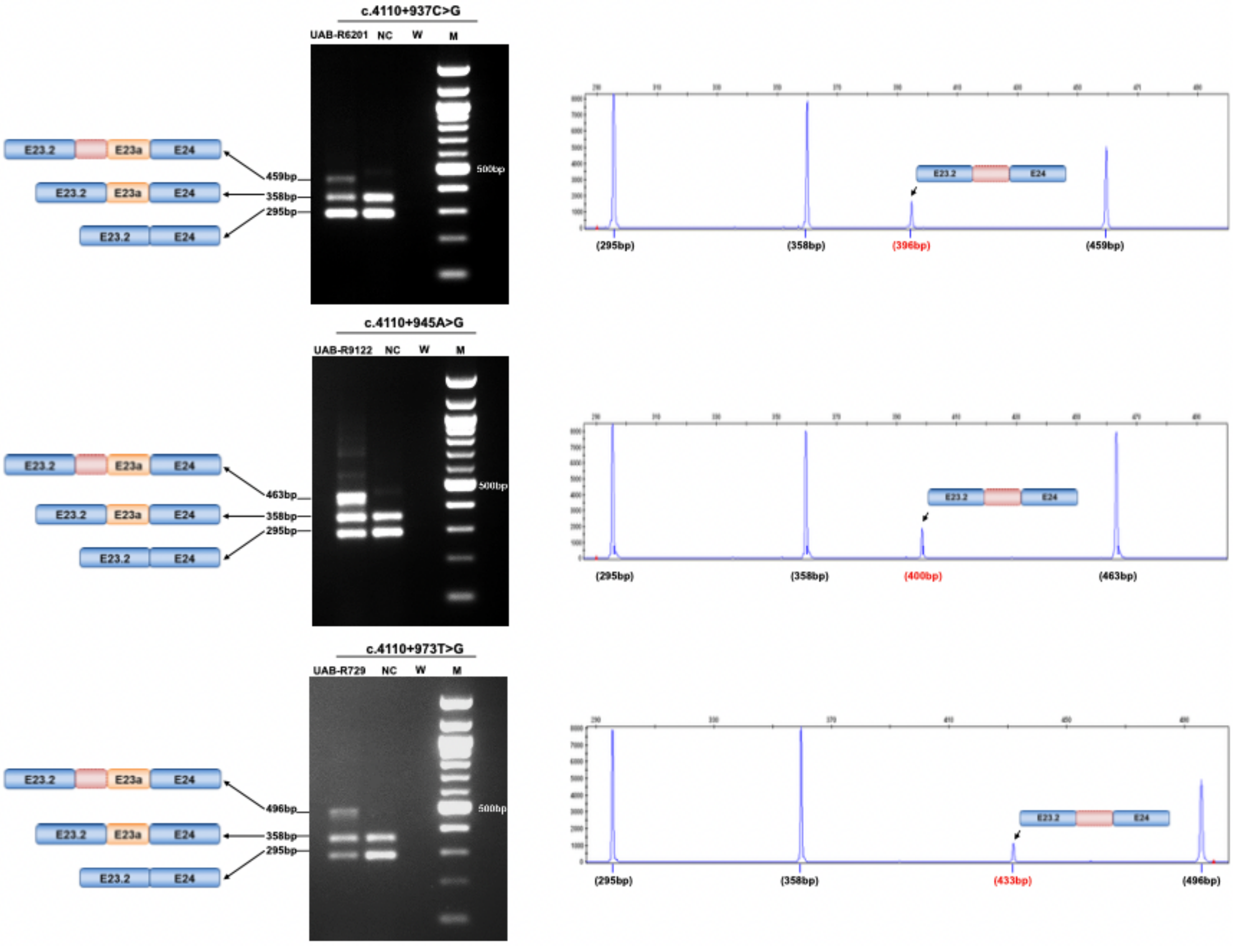

Figure S3. The splicing patterns caused by the *NF1* c.4110+937C>G, c.4110+945A>G and c.4110+973T>G deep intronic variants.

These variants created or activated cryptic splice donor sites and utilized the same splice acceptor site to exonize the intronic sequences from the intron 30 [23.2]. The major aberrant splicing products related to the *NF1* transcript II were observed in the agarose gels as well as the fragment analysis.

Abbreviation: NC - negative control; W - water control; M - marker

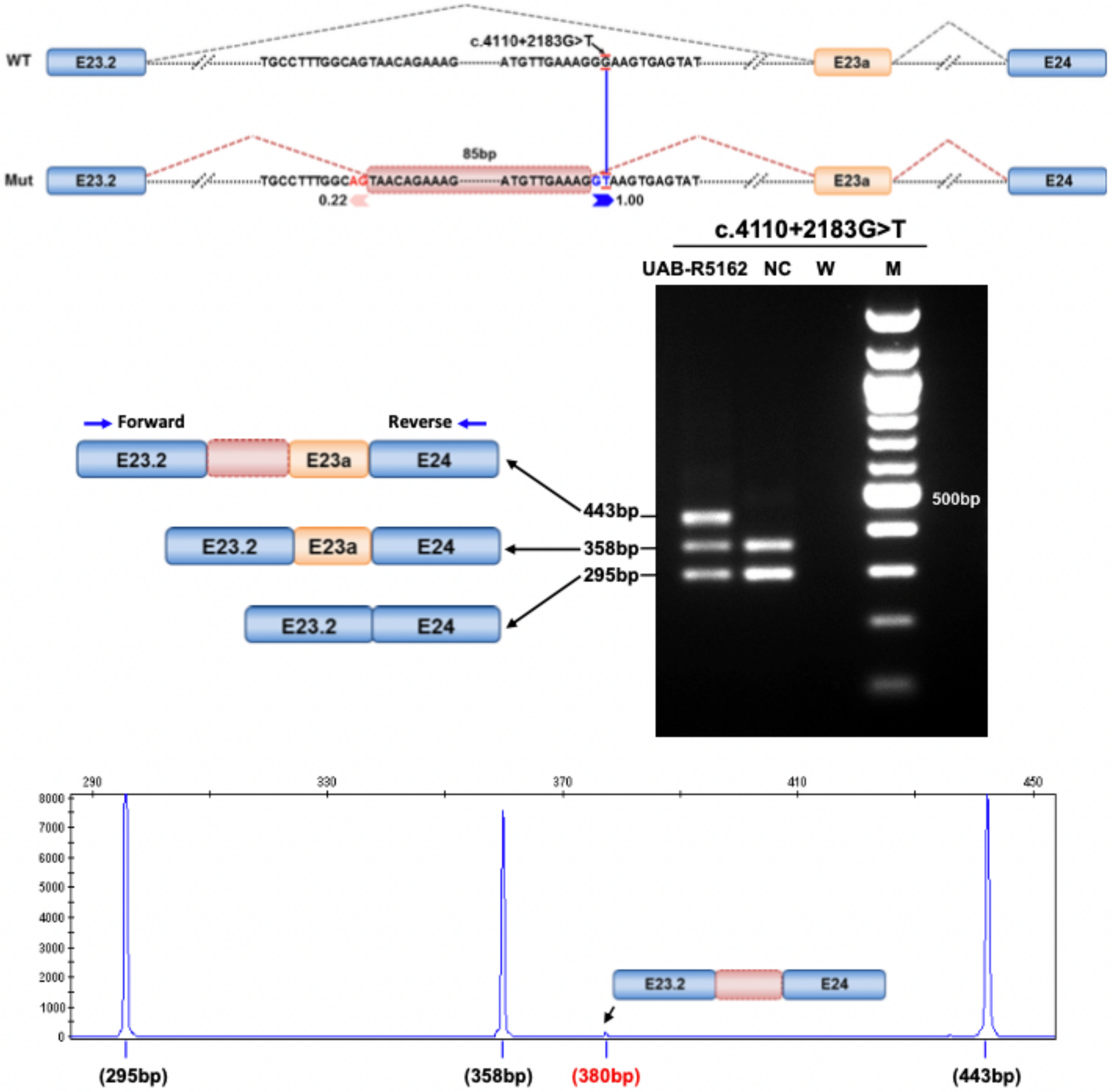

WT
Mut
E23.2
E23a
E24
c.4110+2183G>T
TGCCTTTGGCAGTAACAGAAAG
ATGTTGAAAGGGAAGTGAGTAT
TGCCTTTGGCAGTAACAGAAAG
ATGTTGAAAGGTAAGTGAGTAT
85bp
0.22
1.00
c.4110+2183G>T
UAB-R5162
NC
W
M
Forward
Reverse
443bp
358bp
295bp
500bp
(295bp)
(358bp)
(380bp)
(443bp)

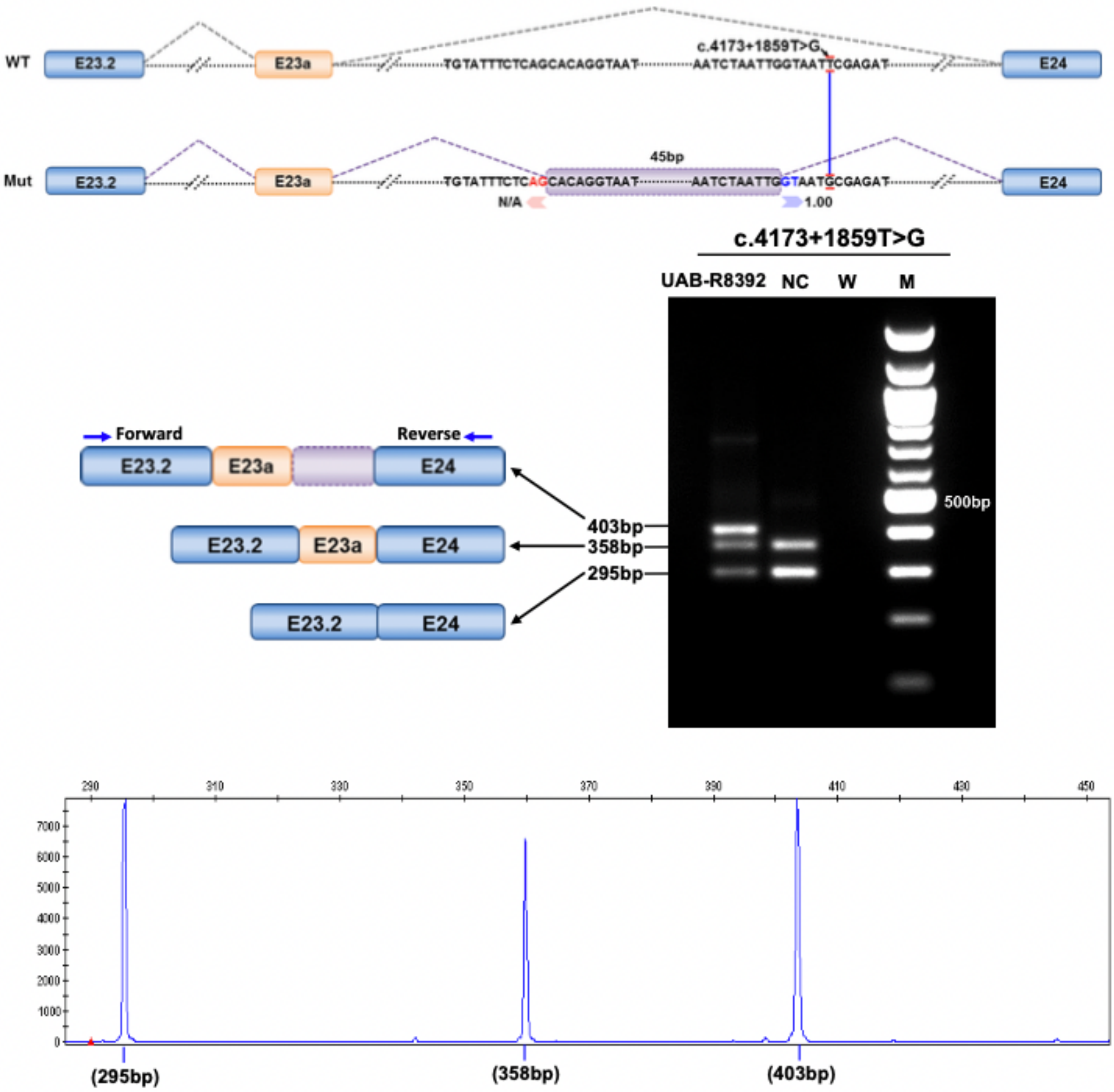

WT
E23.2
E23a
TGTATTTCTCAGCACAGGTAAT
c.4173+1859T>G
AATCTAATTGGTAATTCGAGAT
E24
Mut
E23.2
E23a
45bp
TGTATTTCTCAGCACAGGTAAT
AATCTAATTGGTAATGCGAGAT
E24
N/A
1.00
c.4173+1859T>G
UAB-R8392
NC
W
M
500bp
Forward
Reverse
E23.2
E23a
E24
403bp
358bp
295bp
(295bp)
(358bp)
(403bp)

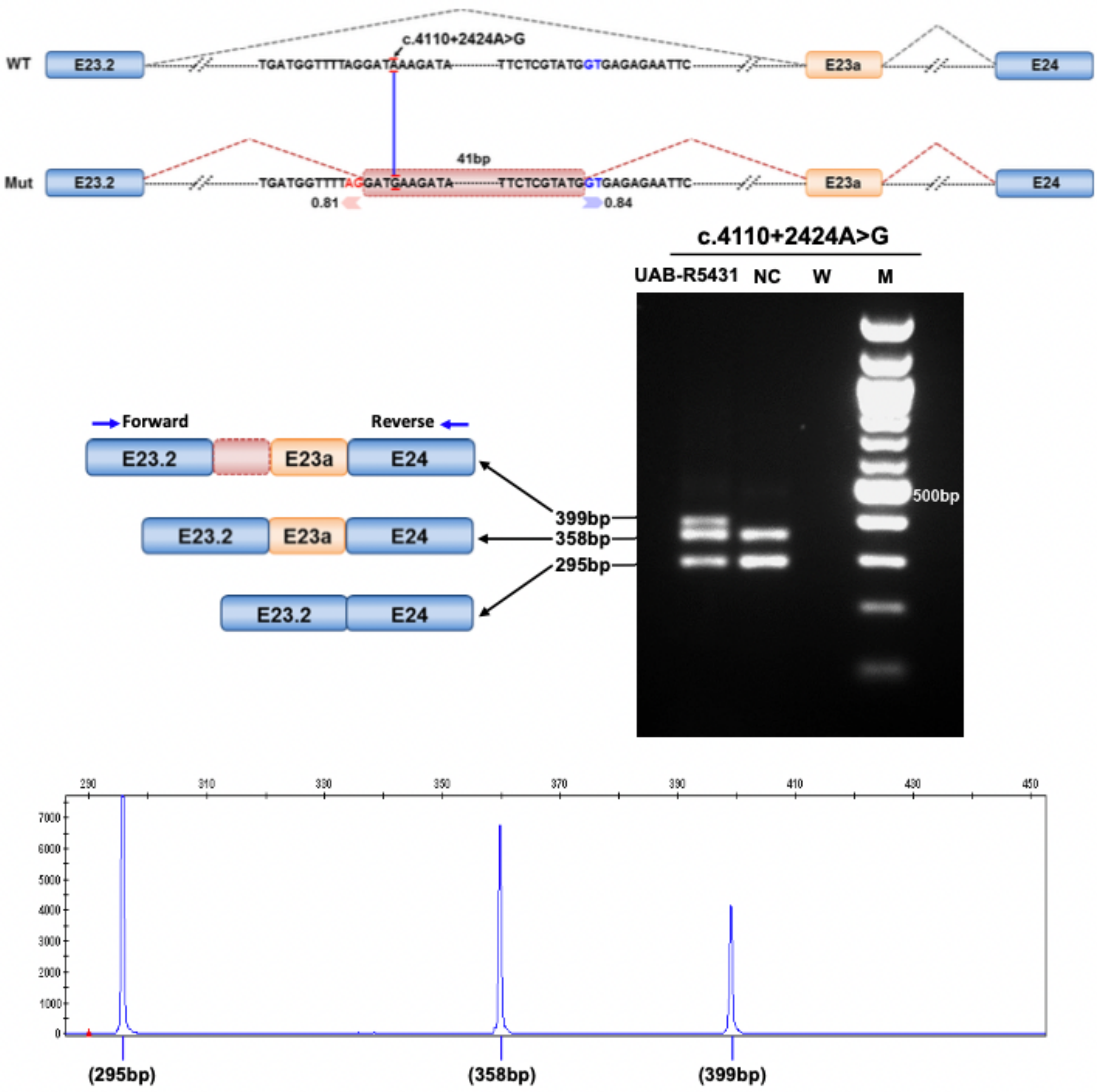

WT
E23.2
TGATGGTTTTAGGATAAAGATA
TTCTCGTATGGTGAGAGAATTC
c.4110+2424A>G
E23a
E24
Mut
E23.2
TGATGGTTTTAGGATGAAGATA
TTCTCGTATGGTGAGAGAATTC
41bp
0.81
0.84
E23a
E24
c.4110+2424A>G
UAB-R5431
NC
W
M
500bp
Forward
Reverse
E23.2
E23a
E24
399bp
358bp
295bp
(295bp)
(358bp)
(399bp)

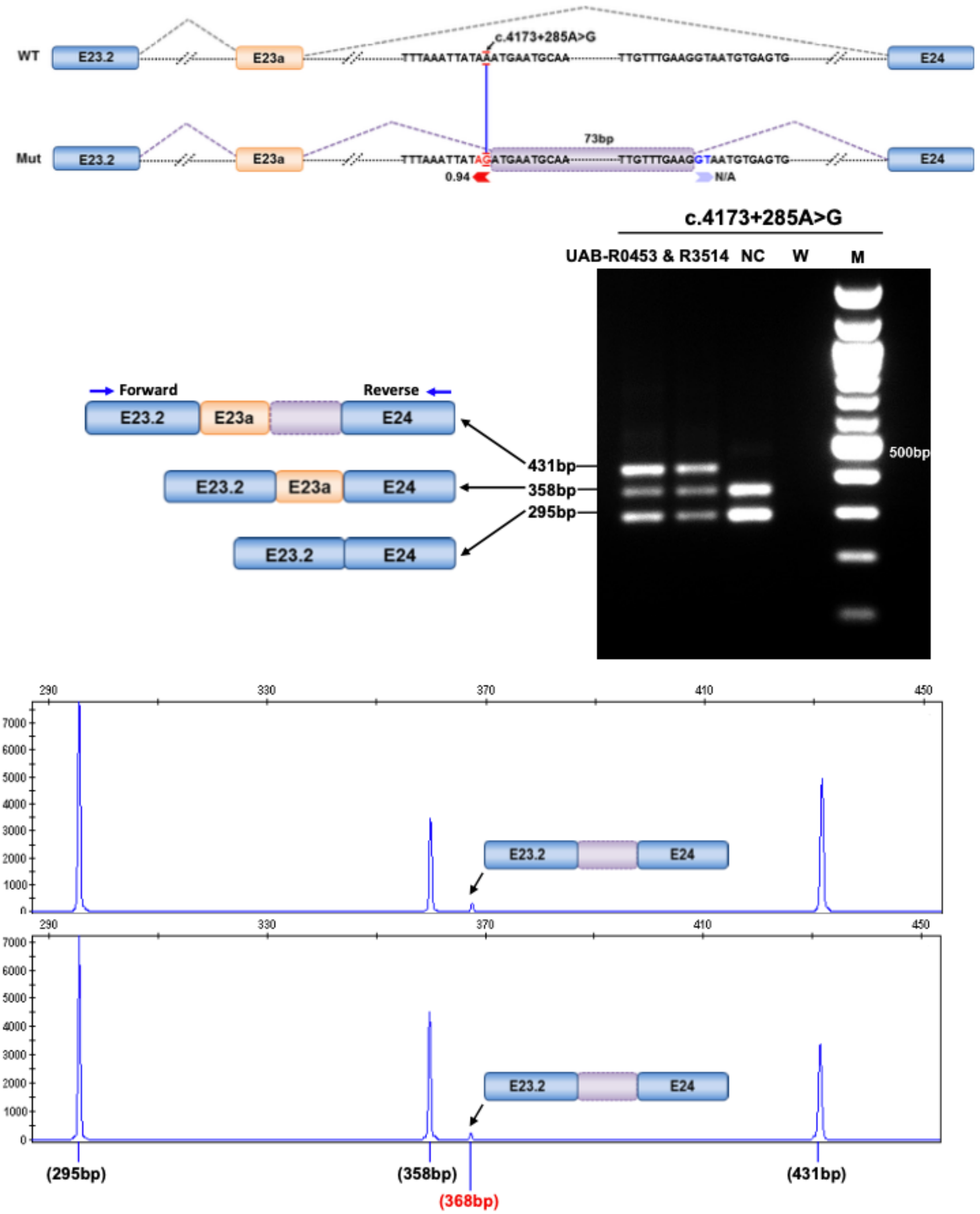

WT
E23.2
E23a
c.4173+285A>G
TTTAAATTATAAATGAATGCAA
TTGTTTGAAGGTAATGTGAGTG
E24
Mut
E23.2
E23a
73bp
TTTAAATTATAGATGAATGCAA
TTGTTTGAAGGTAATGTGAGTG
E24
0.94
N/A
c.4173+285A>G
UAB-R0453 & R3514
NC
W
M
500bp
Forward
Reverse
E23.2
E23a
E24
431bp
358bp
295bp
(295bp)
(358bp)
(368bp)
(431bp)

Figure S4. Characterization of the selected *NF1* deep intronic variants and its effect on the splicing patterns.

This is a schematic presentation showing the location and the flanking sequences of the selected deep intronic variants and the comparison of the splicing patterns between the wild-type and the variant alleles. The cryptic splice acceptor sites and donor sites are highlighted with red and blue. The arrows and the scores underneath indicate the strength prediction of the splice site by NNSPLICE. The light red and blue arrows represent the existed cryptic splice sites, and the dark red and blue arrows represent the *de novo* splice site created by the deep intronic variants. The agarose gels show the cDNA amplification results from the patients. The primers located in exon 30 [23.2] and exon 32 [24] are indicated as line arrows. Each PCR product observed from the agarose gel is also indicated with the size and the schematic composition on the left hand. In the results of the fragment analysis, all the peaks are indicated with the size. For the minor/low level peaks, the size of the peak is highlighted with red color, as well as the schematic composition of the product is presented on top of the peak.

The variant **c.4110+2183G>T** located in the intron 30 [23.2] created a *de novo* splice donor site with 1.00 predicted strength. It utilizes a cryptic splice acceptor site upstream to exonize an 85 bp intronic sequence. On the agarose gel, the largest fragments of 443 bp were derived from the variant allele, which inserted a 85 bp cryptic exon into the *NF1* transcript II including the exon 31 [23a]. In the fragment analysis, an additional minor peak of 380 bp was also observed, which was derived from the mis-splicing of the 85 bp cryptic exon into the *NF1* transcript I excluding the exon 31 [23a], confirmed by TOPO-cloning (data not shown).

The variant **c.4173+1859T>G** located in the intron 31 [23a] activated a cryptic splice donor site with 1.00 predicted strength. It utilized a cryptic splice acceptor site to insert a 45 bp cryptic exon into the transcripts type II.

The variant **c.4110+2424A>G** located in the intron 30 [23.2] activated a cryptic splice acceptor site with 0.81 predicted strength. It utilizes a cryptic splice donor site downstream to exonize a 41 bp intronic sequence into the transcripts. The products of 295 bp and 358 bp were amplified from the normal alleles with or without the alternatively spliced exon 31 [23a]. The largest product of 399 bp was derived from the variant allele, which inserted a 41 bp cryptic exon into the *NF1* transcript II including the exon 31 [23a].

The variant **c.4173+285A>G** located in the intron 31 [23a] created a *de novo* splice acceptor site with 0.94 predicted strength. It utilized a cryptic splice donor site to insert a 73 bp cryptic exon into the transcripts. In the fragment analysis, a minor 368 bp peak was observed, which was derived from the mis-splicing of the 73 bp cryptic exon into the *NF1* transcript I excluding the exon 31 [23a].

The identification and characterization of the c**.4173+278A>G** variant has previously been described in Kannu et al. (2013).

Abbreviation: NC - negative control; W - water control; M - marker

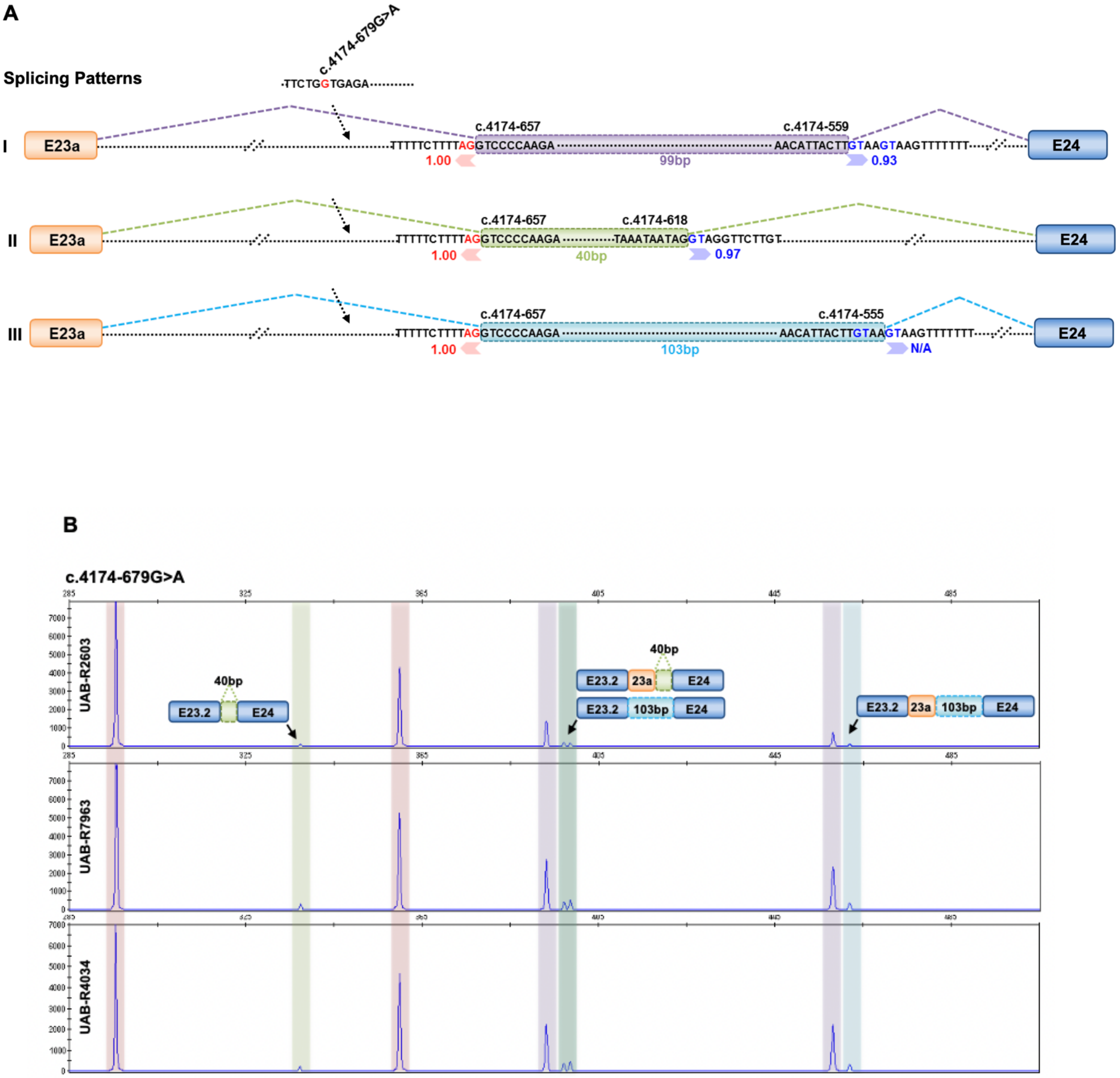
A
Splicing Patterns
c.4174-679G>A
TTCTGGTGAGA
I
E23a
c.4174-657
c.4174-559
TTTTTCTTTTAGGTCCCCAAGA
AACATTACTTGTAAGTAAGTTTTTTT
1.00
99bp
0.93
E24
II
E23a
c.4174-657
c.4174-618
TTTTTCTTTTAGGTCCCCAAGA
TAAATAATAGGTAGGTTCTTGT
1.00
40bp
0.97
E24
III
E23a
c.4174-657
c.4174-555
TTTTTCTTTTAGGTCCCCAAGA
AACATTACTTGTAAGTAAGTTTTTTT
1.00
103bp
N/A
E24
B
c.4174-679G>A
UAB-R2603
UAB-R7963
UAB-R4034
40bp
E23.2
E24
23a
103bp

C

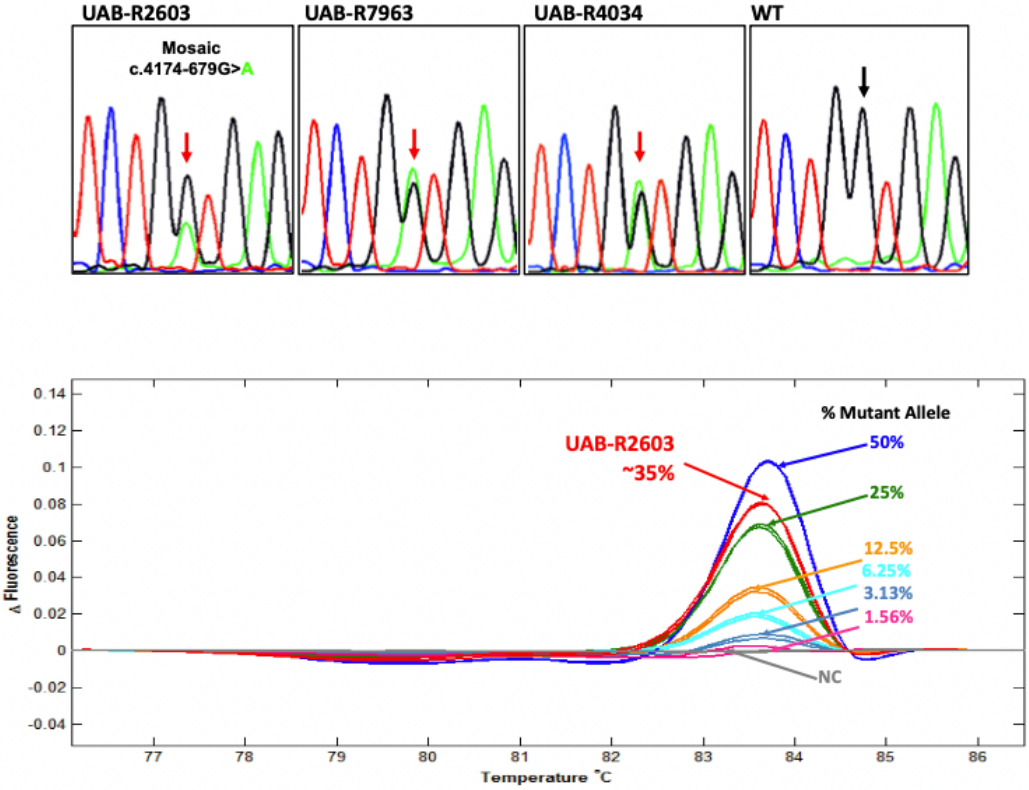

Figure S5. The complex splicing patterns caused by c.4174-679G>A.

A. The schematic distribution of the variants is shown along the intron 31 [23a]. The major cryptic exon of 99 bp caused by the variants is indicated by the purple box with dash outlines. The additional two minor cryptic exons of 40 bp and 103 bp are indicated by the green and light blue boxes with dash outlines. The red and blue arrows indicate the cryptic splice acceptor and donor sites, and the scores are the splice predictions by NNSplice.

B. The fragment analysis results. The peaks highlighted with red color indicate the products of 295 bp and 358 bp, which are derived from the normal allele excluding or including the exon 31 [23a]. The peaks highlighted with purple color indicate the products of 394 bp and 457 bp, which are derived from the *NF1* transcript I and II mis-spliced with the 99 bp cryptic exon. The peaks highlighted with light green color indicate the products of 335 bp and 398 bp, which are derived from the *NF1* transcript I and II mis-spliced with the 40 bp cryptic exon. The peaks highlighted with light blue color indicate the products of 398 bp and 461 bp, which are derived from the *NF1* transcript I and II mis-spliced with the 103 bp cryptic exon. Each peak is also indicated by the schematic composition of the product.

C: Sanger sequencing results of the patients UAB-R2603, UAB-R7963 and UAB-R4034 and the negative control. The variant identified in the patient UAB-R2603 suggests mosaicism. The lower panel shows the melting curve analysis result. The calibration curves are made by a series dilution of the mutant DNA by the wild-type DNA from 50% mutant allele down to 1.56% mutant allele out of all the alleles. The melting curves derived from the UAB-R2603 are indicated in red color, which suggest that the mosaic variant c.4174-679G>A is present in ~35% alleles.

SUPPLEMENTAL REFERENCES

1. Abramowicz A, Gos M (2018). Splicing mutations in human genetic disorders: examples, detection, and confirmation. J Appl. Genet. 59:253-268.
2. Ars E, Kruyer H, Morell M, Pros E, Serra E, Ravella A, Estivill X, Lazaro C (2003) Recurrent mutations in the *NF1* gene are common among neurofibromatosis type 1 patients. J. Med. Genet. 40:e82.
3. Balakrishna T, Curtis D (2020) Assessment of potential clinical role for exome sequencing in schizophrenia. Schizophr. Bull. 46:328-335.
4. Bianchessi D, Morosini S, Saletti V, Ibba MC, Natacci F, Esposito S, Cesaretti C, Riva D, Finocchioro G, Eoli M (2015) 126 novel mutations in Italian patients with neurofibromatosis type 1. Mol. Genet. Genomic Med. 3:513-525.
5. Brinckmann A, Mischung C, Bassmann I, Kuhnisch J, Schuelke M, Tinschert S, Nurnberg P (2007) Detection of novel NF1 mutations and rapid mutation prescreening with Pyrosequencing. Electrophoresis. 28:4295-4301.
6. Carmen Valero M, Martin Y, Hernandez-Imaz E, Hernandez AM, Melean G, Valero AM, Rozdriguez-Alvarez FJ, Telleria D, Hernandez-Chico C (2011) A highly sensitive genetic protocol to detect *NF1* mutations. J. Mol. Diagn. 13:113-122.
7. Castellanos E, Rosas I, Negro A, Gel B, Alibes A, Baena N, Pineda M, Pi G, Pintos G, Salvador H, et al (2020) Mutational spectrum by phenotype: panel-based NGS testing of patients with clinical suspicion of RASopathy and children with multiple café-au-lait macules. Clin. Genet. 97:264-275.
8. Duat Rodriguez A, Martos Moreno GA, Martin Santo-Domingo Y, Hernandez Martin A, Espejo-Saavedra Roca JM, Ruiz-Falco Rojas ML, Argente J (2015) Phenotypic and genetic features in neurofibromatosis type 1 in children. An. Pediatr. (Barc) 83:173-182.
9. Evans DG, Bowers N, Burkitt-Wright E, Miles E, Garg S, Scott-Kitching V, Penman-Splitt M, Dobbie A, Howard E, Ealing J, et al (2016) Comprehensive RNA analysis of the *NF1* gene in classically affected NF1 affected individuals meeting NIH criteria has high sensitivity and mutation negative testing is reassuring in isolated cases with pigmentary features only. EBioMedicine 7:212-220.
10. Fahsold R, Hoffmeyer S, Mischung C, Gille C, Ehlers C, Kucukceylan N, Abdel-Nour M, Gewies A, Peters H, Kaufmann D, et al (2000) Minor lesion mutational spectrum of the entire *NF1* gene does not explain its high mutability but points to a functional domain upstream of the GAP-related domain. Am. J. Hum. Genet. 66:790-818.
11. Fernandez-Rodriguez J, Castellsague J, Benito L, Benavente Y, Capella G, Blanco I, Serra E, Lazaro C (2011) A mild neurofibromatosis type 1 phenotype produced by the combination of the benign nature of a leaky *NF1*-splice mutation and the presence of a complex mosaicism. Hum. Mutat. 32:705-709.
12. Giugliano T, Santoro C, Torella A, Del Vecchio Blanco F, Grandone A, Onore ME, Melone MAB, Straccia G, Melis D, Piccolo V, et al (2019) Clinical and genetic findings in children with neurofibromatosis type 1, Legius syndrome, and other related neurocutaneous disorders. Genes (Basel) 10:580.
13. Jang MA, Kim YE, Kim SK, Lee MK, Kim JW, Ki CS (2016) Identification and characterization of NF1 splicing mutations in Korean patients with neurofibromatosis type 1. J. Hum. Genet. 61:705-709.
14. Jeong SY, Park SJ, Kim HJ (2006) The spectrum of *NF1* mutations in Korean patients with neurofibromatosis type 1. J. Korean Med. Sci. 21:107-112.

15. Kannu P, Nour M, Irving M, Xie J, Loder D, Lai J, Islam O, MacKenzie J, Messiaen L (2013) Paraspinal ganglioneuroma in the proband of a large family with mild cutaneous manifestations on NF1, carrying a deep *NF1* intronic mutation. Clin. Genet. 83:191-194.
16. Legius E, Messiaen L, Wolkenstein P, Pancza P, Avery RA, Berman Y, Blakekey J, Babovic-Vuksanovic D, Cunha KS, Ferner R, et al (2021) Revised diagnostic criteria for neurofibromatosis type 1 and Legius syndrome: an international consensus recommendation. Genet. Med. 23:1506-1513
17. Melloni G, Eoli M, Cesaretti C, Bianchessi D, Ibba MC, Esposito S, Scuvera G, Morcaldi G, Micheli R, Piozzi E, et al (2019) Risk of optic pathway glioma in neurofibromatosis type 1: no evidence of genotype-phenotype correlations in a large independent cohort. Cancers (Basel) 11:1838.
18. Messiaen LM, Wimmer K (2008) Mutational spectrum. D.L. Kaufman (Ed.), Neurofibromates, Basel, Switzerland: Krager, pp. 63-77.
19. Momozawa Y, Iwasaki Y, Parsons MT, Kamatani Y, Takahashi A, Tamura C, Katagiri T, Yoshida T, Nakamura S, Sugano K, et al (2018) Germline pathogenic variants of 11 breast cancer genes in 7,051 Japanese patients and 11,241 controls. Nat. Commun. 9:4083.
20. National Institutes of Health Consensus Development Conference (1988). Neurofibromatosis. Conference statement Arch. Neurol. 45:1355-1381.
21. Osborn MJ, Upadhyaya M (1999) Evaluation of the protein truncation test and mutation detection in the *NF1* gene: mutational analysis of 15 known and 40 unknown mutations. Hum Genet. 105:327-332.
22. Perrin G, Morris MA, Antonarakis SE, Bolthauser E, Hutter P (1996) Two novel mutations affecting mRNA splicing of the neurofibromatosis type 1 (NF1) gene. Hum. Mutat. 7:172-175.
23. Ponti G, Losi L, Martorana D, Priola M, Boni E, Pollio A, Neri TM, Seidenari S (2011) Clinico-pathological and biomolecular findings in Italian patients with multiple cutaneous neurofibromas. Hered. Cancer Clin. Pract. 9:6.
24. Pros E, Gomez C, Martin T, Fabregas P, Serra E, Lazaro C (2008) Nature and mRNA effect of 282 different *NF1* point mutations: focus on splicing alterations. Hum. Mutat. 29:E173-E193.
25. Raponi M, Upadhyaya M, Baralle D (2006) Functional splicing assay shows a pathogenic intronic mutation in neurofibromatosis type 1 (NF1) due to intronic sequence exonization. Hum Mutat. 27:294-295.
26. Richards S, Aziz N, Bale S, Bick D, Das S, Gastier-Foster J, Grody WW, Hegde M, Lyon E, Spector E, et al (2015) Standards and guidelines for the interpretation of sequence variants: a joint consensus recommendation of the American College of Medical Genetics and Genomics and the Association for Molecular Pathology. Genet. Med. 17:405-424.
27. Sabbagh A, Pasmant E, Imbard A, Luscan A, Soares M, Blanche H, Laurendeau I, Ferkal S, Vidaud M, Pinson S, et al (2013) NF1 molecular characgterization and neurofibromatosis type 1 genotype-phenotype correlation: the French experience. Hum Mutat. 34:1510-1518.
28. Spits C, De Rycke M, Van Ranst N, Joris H, Verpoest W, Lissens W, Devroey P, Van Steirteghem A, Liebaers I, Sermon K (2005) Preimplantation genetic diagnosis for neurofibromatosis type 1. Mol Hum Reprod. 11:381-387.
29. Svaasand EK, Engebretsen LF, Ludvigsen T, Brechan W, Sjursen W (2015) A novel deep intronic mutation introducing a cryptic exon causing neurofibromatosis type 1 in a family with highly variable phenotypes: a case study. Hereditary Genetics, 4:3.

30. Terzi YK, Oguzkan-Balci S, Anlar B, Varan A, Ersoy-Evans S, Sharafi P, Ayter S (2018) Clinical findings and mutation analysis of NF1 patients in Turkey. Meta Gene 15:80-83.
31. Wang X, Kallionpaa RA, Gonzales PR, Chitale DA, Tousignant RN, Crowley JP, Chen Z, Yoder SJ, Blakeley JO, Acosta MT et al (2018) Germline and somatic *NF1* alterations are linked to increased HER2 expression in breast cancer. Cancer Prev. Res. (Phila) 11:655-664.
32. Wimmer K, Roca X, Beiglbock H, Callens T, Etzler J, Rao AR, Krainer AR, Fonatsch C, Messiaen L (2007) Extensive in silico analysis of Nf1 splicing defects uncovers determinants for splicing outcome upon 5' splice-site disruption. Hum. Mutat. 28:599-612.
33. Wimmer K, Schamschula E, Wernstedt A, Traunfellner P, Amberger A, Zschocke J, Kroisel P, Chen Y, Callens T, Messiaen L (2020). AG-exclusion zone revisited: Lessons to learn from 91 intronic NF1 3'splice site mutations outside the canonical AG-dinucleotides. Hum. Mutat. 41:1145-1156.
